# Flexibility of equine bioenergetics and muscle plasticity in response to different types of training: An integrative approach, questioning existing paradigms

**DOI:** 10.1371/journal.pone.0249922

**Published:** 2021-04-13

**Authors:** Constance de Meeûs d’Argenteuil, Berit Boshuizen, Maarten Oosterlinck, Don van de Winkel, Ward De Spiegelaere, Cornelis Marinus de Bruijn, Klara Goethals, Katrien Vanderperren, Cathérine John Ghislaine Delesalle

**Affiliations:** 1 Department of Virology, Parasitology and Immunology, Research Group of Comparative Physiology, Faculty of Veterinary Medicine, Ghent University, Merelbeke, Belgium; 2 Wolvega Equine Hospital, Oldeholtpade, The Netherlands; 3 Department of Surgery and Anaesthesiology of Domestic Animals, Faculty of Veterinary Medicine, Ghent University, Merelbeke, Belgium; 4 Department of Morphology, Faculty of Veterinary Medicine, Ghent University, Merelbeke, Belgium; 5 Department of Nutrition, Genetics and Ethology, Research Group Biometrics, Faculty of Veterinary Medicine, Ghent University, Merelbeke, Belgium; 6 Department of Veterinary Medical Imaging and Small Animal Orthopaedics, Faculty of Veterinary Medicine, Ghent University, Merelbeke, Belgium; University of Illinois, UNITED STATES

## Abstract

Equine bioenergetics have predominantly been studied focusing on glycogen and fatty acids. Combining omics with conventional techniques allows for an integrative approach to broadly explore and identify important biomolecules. Friesian horses were aquatrained (n = 5) or dry treadmill trained (n = 7) (8 weeks) and monitored for: evolution of muscle diameter in response to aquatraining and dry treadmill training, fiber type composition and fiber cross-sectional area of the M. pectoralis, M. vastus lateralis and M. semitendinosus and untargeted metabolomics of the M. pectoralis and M. vastus lateralis in response to dry treadmill training. Aquatraining was superior to dry treadmill training to increase muscle diameter in the hindquarters, with maximum effect after 4 weeks. After dry treadmill training, the M. pectoralis showed increased muscle diameter, more type I fibers, decreased fiber mean cross sectional area, and an upregulated oxidative metabolic profile: increased β-oxidation (key metabolites: decreased long chain fatty acids and increased long chain acylcarnitines), TCA activity (intermediates including succinyl-carnitine and 2-methylcitrate), amino acid metabolism (glutamine, aromatic amino acids, serine, urea cycle metabolites such as proline, arginine and ornithine) and xenobiotic metabolism (especially p-cresol glucuronide). The M. vastus lateralis expanded its fast twitch profile, with decreased muscle diameter, type I fibers and an upregulation of glycolytic and pentose phosphate pathway activity, and increased branched-chain and aromatic amino acid metabolism (cis-urocanate, carnosine, homocarnosine, tyrosine, tryptophan, p-cresol-glucuronide, serine, methionine, cysteine, proline and ornithine). Trained Friesians showed increased collagen and elastin turn-over. Results show that branched-chain amino acids, aromatic amino acids and microbiome-derived xenobiotics need further study in horses. They feed the TCA cycle at steps further downstream from acetyl CoA and most likely, they are oxidized in type IIA fibers, the predominant fiber type of the horse. These study results underline the importance of reviewing existing paradigms on equine bioenergetics.

## Introduction

Equestrian sports competition takes place with an ever-increasing frequency and intensity, even at the recreational and semi-professional level. To prevent the occurrence of sports injuries, the application of a thorough and well-considered training protocol is of utmost importance. The purpose of a well-considered training protocol is threefold: 1) creating stamina or aerobic capacity, which is the basis for any form of performance capacity; 2) practicing of specific skills such as racing, show jumping, dressage etc.; and 3) ensuring that different parts of the athlete’s body adapt to the competition type and level at which it needs to perform [1–3]. The latter adaptation is seen for example in the bony skeleton, strengthening itself in response to training load and also in specific muscle groups that show plasticity and thus physiologically adapt in response to specific types of training [4–7]. This adaptation manifests itself mainly at three different levels within the muscle. First of all, it is well known that shifts in muscle fiber type composition occur as a consequence of certain types of training [7–15]. Associated with that, muscle groups can either increase or decrease in muscle mass. Ideally, these adaptations are ultimately seen in the main muscle groups responsible for force and locomotion necessary for a certain sports discipline. Since each of these muscle fiber types uses its own specific set of main metabolic pathways, shifts also take place in the metabolic fingerprint of muscle groups in response to training [16–23]. On top of that, not all muscles show the same adaptation in response to a certain type of training. Muscle groups that are predominantly involved in posture will show a different adaptation pattern when compared to muscle groups that are primarily involved in locomotion. However, up until now, no equine studies are available that apply a standardized multimodal approach looking into the effect of different types of training on changes in muscle diameter, muscle fiber type composition and muscle bioenergetics of a multitude of muscles and also providing a view on when the maximal training effect is to be expected. The strategic combination of novel “omics” techniques with more conventional analysis techniques allows for exploring the possible existence of previously unknown pathways and candidate fuels and to evaluate their importance. Many equine energy metabolism studies have been focusing on knowledge extrapolated from human and ruminant studies [24, 25]. However, horses are hind-gut fermenters, so, differences from both human and ruminant energy metabolism are to be expected. By monitoring the evolution of the muscle diameter in a set of 15 strategically chosen muscles by morphometric assessment, it becomes possible to obtain a detailed view of the core set of muscles on which each training technique has its focus effect.

Muscle fibers are classified as either slow twitch (type I) fibers or fast twitch (type IIA, type IIX and hybrid type IIAX) fibers. Type I fibers have a small fiber cross-sectional area (CSA), which is associated with a decreased diffusion distance for oxygen transport. These fibers have a high capillary number and rely on rapid supply of fuels through the circulatory system. Moreover, they are fatigue resistant and rely on mainly aerobic metabolism and thus, the electron transfer system as final step for ATP production.

In contrast, type II fibers have a large fiber CSA and thus a high storage capacity for fuels. Type IIA fibers are fast aerobic glycolytic. They realize fast contractions using primarily oxidative pathways. Type IIX and type IIAX muscle fibers represent a transitional form [10, 11, 26–28].

Distribution of fast twitch versus slow twitch fibers in human skeletal muscles on a whole equals approximately a 50% ratio [29, 30]. In horses, fast twitch muscle fibers of type IIA, are the predominant type [31, 32].

Although Friesian horses’ performance capacity has recently been evaluated with Standardized Exercise Tests (SET) [33], studies focusing on muscle fiber type composition of this breed are lacking. Friesian horses are genetically related to cold-blooded draught breeds, such as Haflinger, Dutch draft and Belgian draft, which are heavily muscled breeds that are able to generate high-power output [34, 35].

In essence, the metabolic fingerprint of a certain muscle group needs to be viewed as the compilation of the metabolic fingerprint of all of the individual muscle fibers harbored within that muscle group. Shifts in muscle fiber type composition that occur in response to certain types of training coincide with shifts in the metabolic profile of a certain muscle group [7, 9, 11, 36, 37].

In human sports and training science, a lot of information is available about the effect of different types of training approaches on muscle plasticity and shifts in muscle fiber type composition [7, 9, 11, 14, 15, 26, 36]. In equine sports medicine, the number of studies, comparing plasticity and muscle fiber type composition shifts in response to different types of training is growing rapidly [38–52]. Still, equine studies focusing on shifts in the muscle metabolic fingerprint in response to training are very scarce [23, 53–55]. A few human [56–58], equine [23, 59–62] and rodent [20, 63, 64] studies have looked into shifts in blood metabolomic profiles in response to training. However, the circulation as body compartment connects to all organ systems, such as the liver, gastro intestinal tract, etc., which makes it nearly impossible to link these study results one on one to shifts in muscle metabolism, as has been shown by Zhang et al. [17].

**Fig 1:**
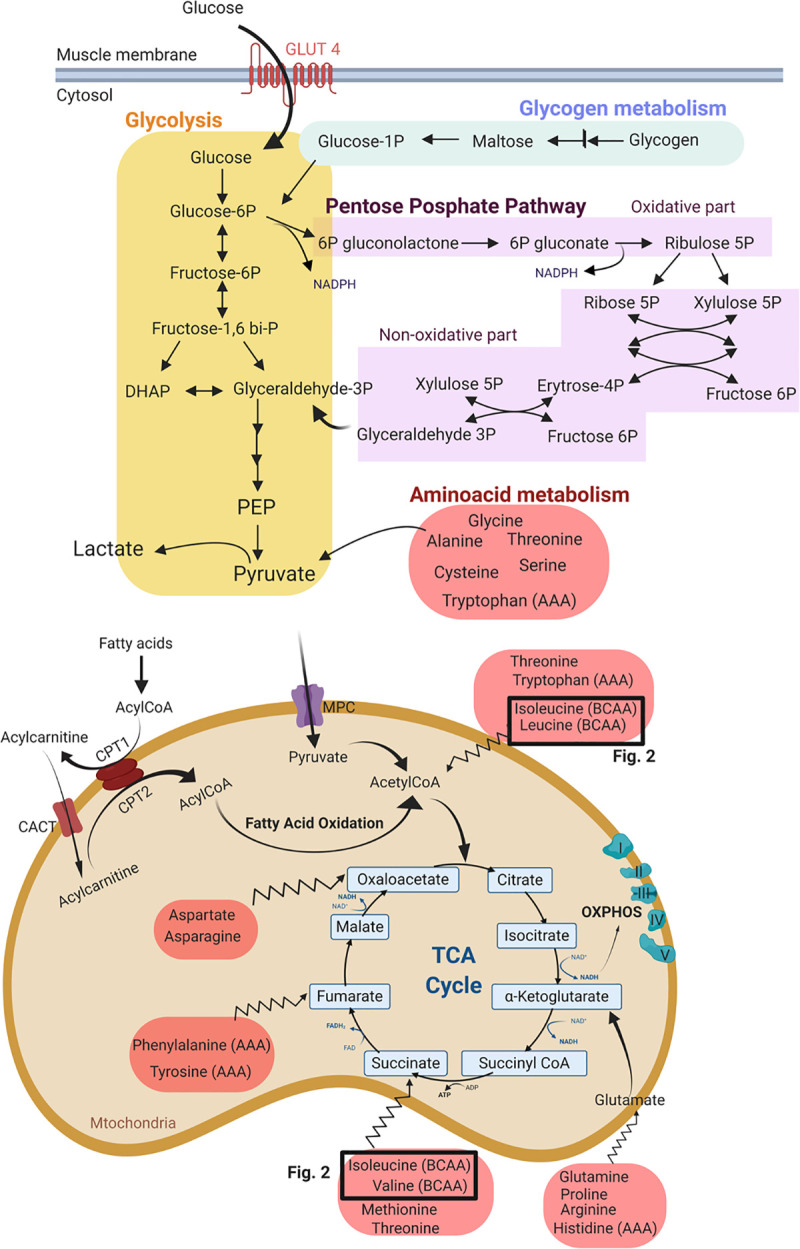
Metabolism in the cytosol and mitochondria of the skeletal muscle. The metabolites are grouped in different pathways: the glycogen metabolism pathway, glycolysis, pentose phosphate pathway (PPP) and amino acid metabolism: BCAA: branched-chain amino acid; AAA: aromatic amino acid; PEP: phosphoenol pyruvic acid; MPC: mitochondrial pyruvate carrier; CPT: carnitine palmitoyl transferase; OXPHOS: oxidative phosphorylation. The TCA cycle is the final and universal step before the vast amount of ATP is created at the level of the electron transport system (OXPHOS). Apart from fat and glucose, also proteins can be catabolized to produce precursors of glycolysis and the TCA cycle. Amino acids can feed into the TCA cycle at acetyl CoA, as well as at steps further downstream from acetyl CoA (jagged arrows).

**Fig 2:**
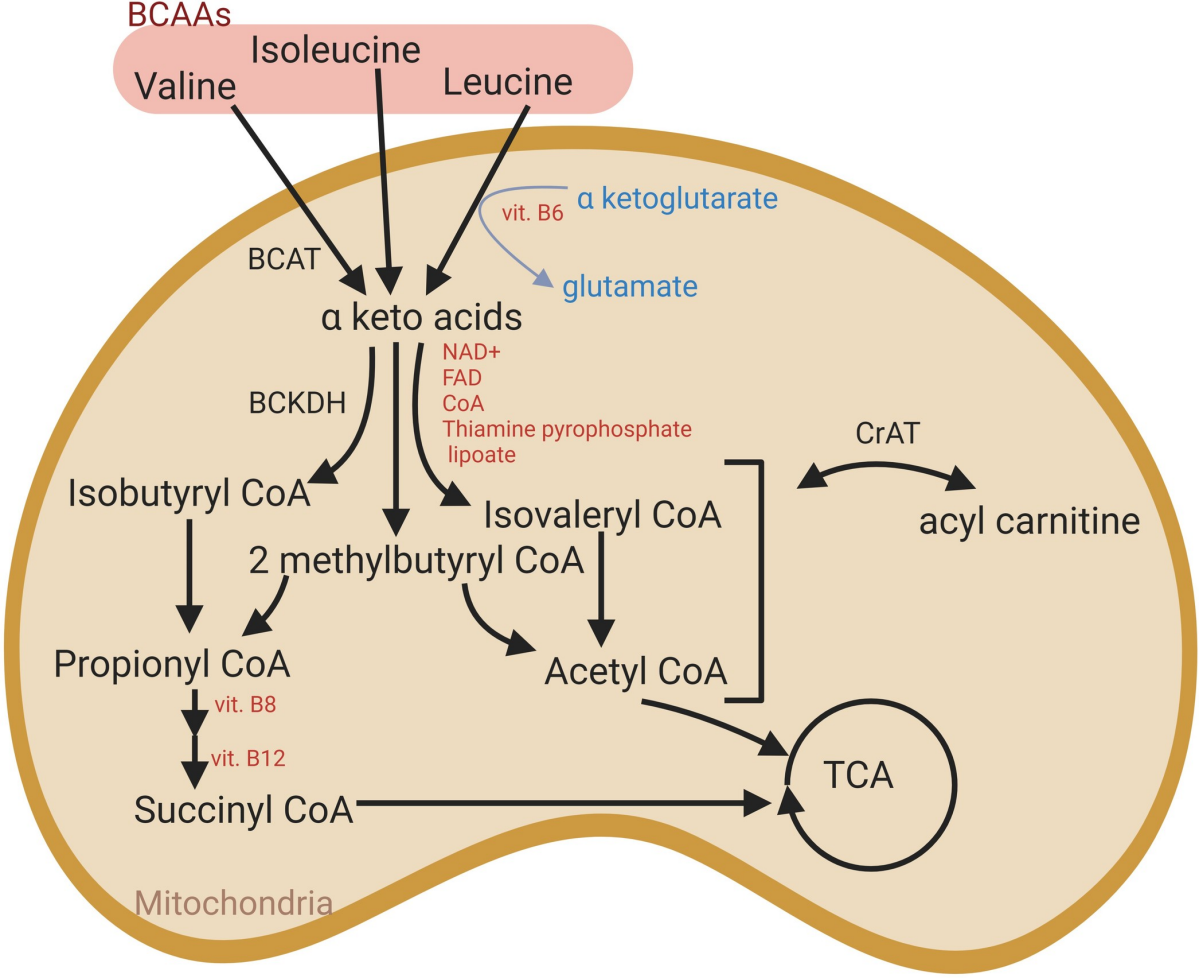
Branched-chain amino acid metabolism (BCAA) in muscle mitochondria. Intermediates derived from alternative pathways can feed into the TCA cycle. BCAT: branched-chain amino acid aminotransferase; BCKDH: branched-chain keto-acid dehydrogenase complex; CrAT: carnitine acetyltransferase.

Most energy cycles were discovered a long time ago, such as the tricarboxylic acid cycle (TCA), which was discovered by Hans Krebs in 1935 (Fig 1). All these energy pathways have been intensively described. Apart from fat and glucose, also proteins can be catabolized to produce precursors of glycolysis and the TCA cycle. Amino acids can feed the TCA cycle at different levels (jagged arrows in Fig 1). Figs 1 and 2 provide an overview of the different energy cycles.

Up until now, especially glycogen and short chain fatty acids (SCFA) have received a lot of attention in equine metabolic studies. However, evidence is accumulating that other important substances might have been overlooked in the past [23, 53, 62]. With that respect, untargeted metabolomics provide a view on previously unexplored substrates.

Obviously, the type of training and training load play a crucial role in the shift that occurs at the level of the muscle fiber type composition and metabolic fingerprint of a specific muscle group and the core set of muscles that is modulated [65–69]. For the current study it was decided to focus on dry treadmill training and aquatraining.

Dry treadmill exercise (DT) is often added to training and rehabilitation protocols in horses, although its effects on different muscle groups and their metabolism is not completely clear [70]. This type of training is often applied in exercise studies since it allows for controlling many parameters such as speed, inclination, duration, environmental conditions, etc. [70]. Once the horse is habituated to this type of training, high constancy in stride variables has been described [71]. It has been shown that DT increases aerobic capacity and improves the cardiovascular function of horses [72, 73].

Water treadmill exercise or aquatraining (AT) is increasingly incorporated into equine training and rehabilitation programs because it combines moderate intensity exercise with minimal burden on tendons and articulations [74, 75]. However, little is known about the physiological adaptive training responses occurring in horses subjected to AT. A few studies have been performed on the effect of AT on aerobic capacity, including the effect of different belt speeds, water heights and water temperatures on physiological parameters such as heart rate, skin temperature and blood lactic acid levels [74–79]. Both human and equine studies have pointed out that AT is an aerobic form of exercise, although it does not seem to increase aerobic capacity in a training protocol [75]. Human, equine and canine studies have shown that water height has a significant effect on kinematic responses [76, 77, 80–84]. However, little effect on heart rate and blood lactic acid levels could be seen during AT in horses (water height from baseline to 80% of the wither height) [76]. Scott et al. (2010) [77] did not see a difference in heart rate when comparing DT to AT. Reported plateaus for lactic acid values and heart rate seen during AT correspond with exercise performed within the aerobic window [74–77]. A recent study tested 3 different speeds (1.11; 1.25; 1.39 m/sec) and water heights (mid-canon, carpus, stifle) on respiratory and cardiovascular parameters in Quarter horses, a breed well known for its richness in type IIX muscle fibers. They concluded that the heart rate was significantly higher in AT horses when compared to DT horses and that this difference became more pronounced with increasing water heights (from mid-canon to stifle) [80].

Up until now, no standardized equine studies are available applying longitudinal follow up of muscle diameter, muscle fiber type composition and untargeted metabolomic fingerprint assessment on specific muscle groups in response to training. Even in human sports medicine such studies are lacking. A targeted metabolic study was performed by Borgia et al. (2010) [55] who found no changes in resting concentrations of gluteal or superficial digital flexor muscle glycogen, lactic acid, ATP or glucose-6-phosphate, or activities of citrate synthase, 3-hydroxyacyl-CoA dehydrogenase and lactate dehydrogenase after 4 weeks of AT when compared to starting conditions in 5 horses of mixed breeds. This was in accordance with a study of Firshman et al. (2015) [40] in which 6 Quarter horses were trained in a cross-over design on a conventional treadmill and then on a deep water treadmill (water up to olecranon; belt speed 1.5 m/sec) for 8 weeks, with 60 days detraining in between. In this study, no training effect could be seen on muscle fiber type composition, nor heart rate, muscle metabolites or blood lactic acid [40]. Recently, an untargeted metabolomics study of the M. gluteus medius of 8 Standardbred horses was performed, looking into the effect of 12 weeks of DT and the effect of acute fatiguing intense exercise on a treadmill [23]. In that study, muscle biopsies and plasma samples were taken before and respectively 3 and 24h after training, at start (unconditioned state) and finish (conditioned state) of the training trial. Klein et al. (2020) [23] reported that DT had significant effects on nucleotide- and xenobiotic related markers and increased almost all long chain fatty acids as well as long chain acylcarnitines and branched-chain amino acid derived acylcarnitines (C3 and C5). Plasma samples showed similar profiles as muscle biopsies when comparing conditioned with unconditioned state, but did not show significant differences when comparing samples before and after acute exercise [23].

### Aims of the study

The aims of the current study were (1) to identify the skeletal muscles that show significant changes in muscle diameter in response to respectively 8 weeks of aquatraining (AT) and 8 weeks of dry treadmill training (DT); and (2) to provide an overview of changes in the muscular bioenergetics, muscular fiber type composition and fiber CSA induced by 8 weeks of DT.

## Material and methods

### Study design

A first group of seven healthy untrained client owned Friesian horses (age range 2.5–3.5 years; 4 ♀ and 3 intact ♂) completed a dry treadmill training program (DT) of 8 weeks duration (20 min per session, 5 days/week, belt speed 1.25 m/sec). A second group of five healthy untrained client owned Friesian horses (age range 2.5–3.5 years; 2 ♀ and 3 intact ♂) completed an 8 weeks aquatraining (AT) program in the same device (20 min per session, 5 days/week, water height: mid-metacarpus, water temperature 7°C, belt speed 1.25 m/sec). Horses were not trained in any way before this study. Both training periods were preceded by 2 weeks of acclimatization and the time, speed and intensity of both training regimens remained constant throughout the study. The same concentrate feed and source of roughage was used throughout both studies in all horses. Horses were fed concentrate feed twice a day, at 8 AM and 8 PM. Horses were housed in individual boxes and did not have access to pasture during the entire trial nor during the acclimatization period. Vital signs were recorded twice a day: rectal temperature, respiratory rate, heart rate, capillary refill time and color of mucous membranes, appetite and fecal consistency. Right before, immediately after and 10 minutes after cessation of each training session the heart rate of each horse was registered by auscultation by the same person. Ethical approval for this study was granted by the Centrale Commissie Dierproeven, The Hague, The Netherlands, file AVD262002015144 and all efforts were made to maximize animal welfare throughout the study.

### Muscle morphometrics

Throughout both training studies, morphometric assessment of 15 strategically chosen muscle groups was performed on 3 different occasions: at start, after 4 weeks and at finish of the training protocol (8 weeks) at both sides of the body, using transcutaneous B-mode ultrasound (Esaote, macroconvex probe, 2.5–4.3 MHz). When needed, horses were sedated using detomidine (10 μg/kg bwt) (Detogesic^®^, Vetcare, Finland) and butorphanol (20 μg/kg bwt) (Butomidor^®^, Richter Pharma AG, Wels, Austria).

**Fig 3:**
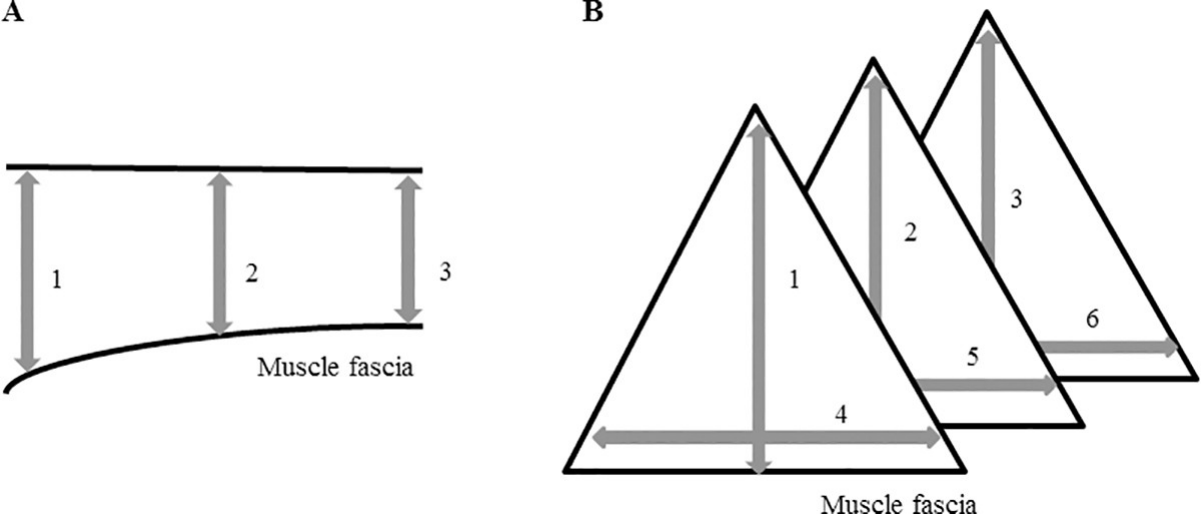
Ultrasonographic assessment of muscle morphometrics. (A) in spindle shaped muscles; (B): in triangular shaped muscles.

The areas of interest were clipped, scrubbed with chlorhexidine digluconate (Hibiscrub^®^, Regent Medical Ltd., Oldham, Lancashire, United Kingdom) and subsequently shaved and covered with ultrasound coupling gel. Shaving was performed on a regular basis throughout the study to assure that ultrasound was always performed on the same anatomical locations. Muscle diameters were compared between left and right body side and throughout the training period, in order to identify muscle groups showing either an increase, a decrease or no change in muscle diameter. The transsectional diameter of each muscle was measured at three different locations for spindle shaped muscles: in the middle, at the origin and the insertion site (Fig 3A) and on 6 different locations for the triangular shaped muscles (M. semimembranosus and M. semitendinosus) (Fig 3B). Each measure was executed twice and the mean was taken. In both studies all ultrasounds were performed by the same certified veterinarian.

### Muscle biopsies

In the DT group, fine needle muscle biopsies were harvested. For the DT group this was performed at start and finish of the study, at rest, on a non-training day, from the M. pectoralis, M. vastus lateralis of the quadriceps femoris and the M. semitendinosus. Briefly, the horses were sedated with detomidine (10 μg/kg bwt) (Detogesic^®^, Vetcare, Finland) and butorphanol (20 μg/kg bwt) (Butomidor^®^, Richter Pharma AG, Wels, Austria). The area was clipped, shaved and subsequently surgically disinfected. Local anesthetic ointment was applied (Emla^®^ 5%, Astra-Zeneca, Rueil-Malmaison, France). After 10 minutes, local anesthetic solution (Lidocaine Hydrochloride^®^, Braun, Germany) was injected subcutaneously and a small stab incision was made with a surgical blade number 11. Subsequently, a 14G Bergström needle was inserted into the muscle, until a depth of 4 cm was reached on each occasion. Two samples were taken under suction pressure, to obtain a total of approximatively 120 mg muscle tissue, which was then divided into three portions: one sample was embedded in Tissue-Tek^®^ OCT compound (Sakura Finetek, Torrance, CA) and was immediately snap-frozen in isopentane in liquid nitrogen and stored at -80°C until processed for muscle fiber typing and fiber CSA assessment. The remaining portions were immediately snap frozen in liquid nitrogen and stored at -80°C until processed for untargeted metabolomics.

### Muscle fiber typing

**Fig 4:**
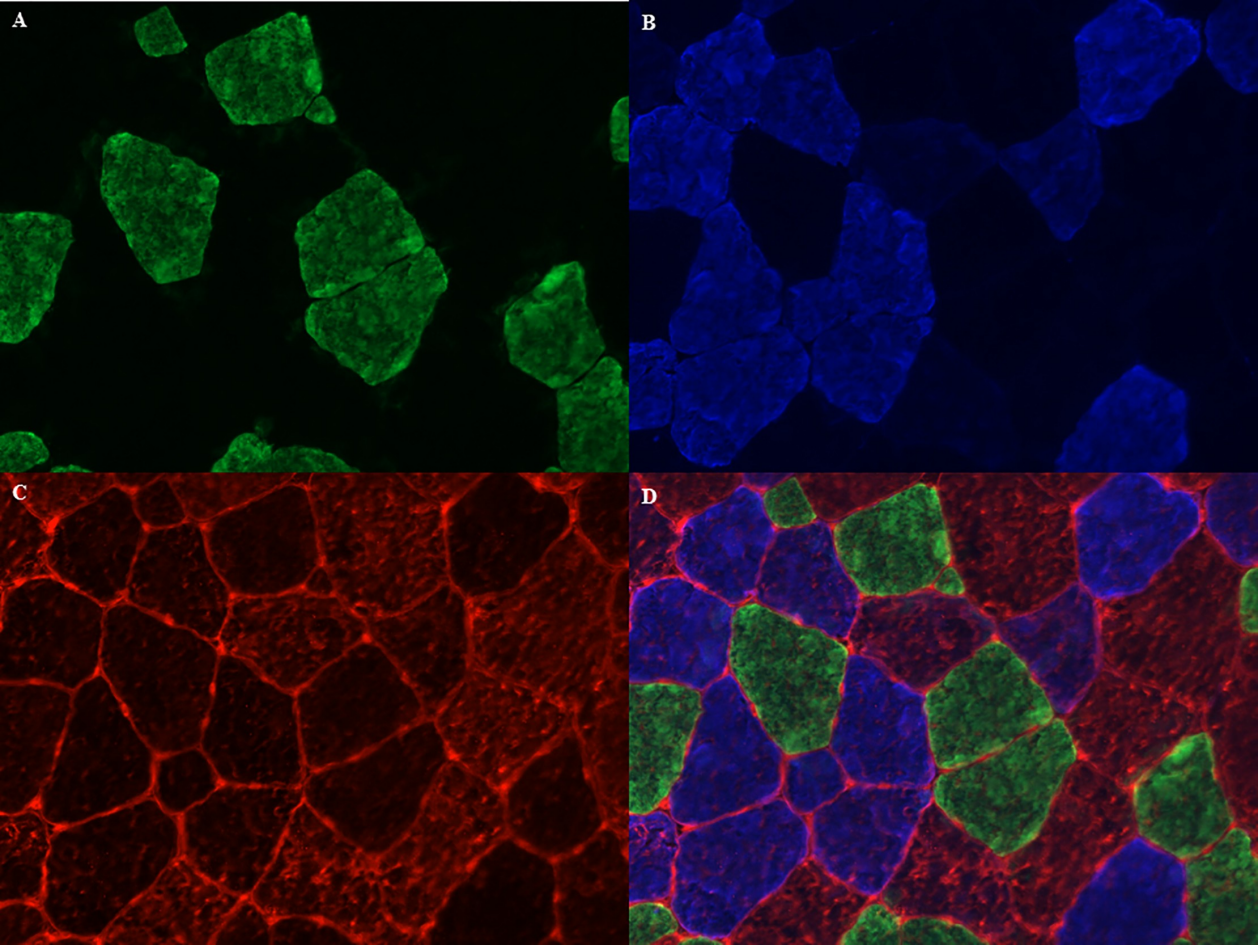
Muscle fiber typing with myosin heavy chain staining method. (A) type I fibers in green; (B) type IIA fibers in blue; (C) type IIX fibers and sarcolemma in red; (D) merged image.

Cryosections of 8 μm were created from the Tissue-Tek^®^ embedded samples from the M. pectoralis, the M. vastus lateralis and the M. semitendinosus and were collected onto Thermo Scientific™ SuperFrost Plus™ Adhesion slides and stored at -20°C until further processing. In brief, the sections were air-dried and then blocked for 120 minutes in 1% BSA in PBS solution. Thereafter, the slides were incubated overnight with the primary antibodies for the different myosin heavy chains, which were validated by Latham & White (2017), for type I, type IIA, type IIX and sarcolemma (respectively BA-D5, DSHB, RRID:AB_2235587; SC-71, DSHB, RRID:AB_2147165; 6H1, DSHB, RRID:AB_1157897 and laminin, Thermo Fisher Scientific Cat: PA1-36119, RRID:AB_2133620) [85]. After rinsing the slides 5 consecutive times during 5 minutes in PBS, they were incubated with the secondary antibodies for 1h at room temperature for type I, IIA, IIX and sarcolemma (respectively: Alexa fluor 488 goat anti mouse IgG2b, Thermo Fisher Scientific Cat: A-21141, RRID:AB_2535778; Alexa fluor 350 goat anti mouse IgG1, Thermo Fisher Scientific Cat: A21120, RRID:AB_2535763; Alexa fluor 594 goat anti mouse IgM, Thermo Fisher Scientific Cat: A-21044, RRID:AB_2535713; Alexa fluor 568 goat anti-rabbit IgG, Thermo Fisher Scientific Cat: A-11011, RRID:AB_143157). Fluorescent mounting medium (Dako, Agilent, S3023) was then applied on the slides. The sections were visualized with a Zeiss Palm Micro Beam fluorescence microscope and pictures were taken with the Zen Blue Pro^®^ Software (Zeiss). On average, 690 fibers were analyzed on each section, with a minimum of 250 fibers and they were classified as type I (green), type IIA (blue), type IIX (red) or as hybrid type when staining for more than one myosin heavy chain was present (Fig 4). In the current study only hybrid type IIA/IIX (IIAX) fibers were included, since the hybrid type I/IIA was only sporadically found (less than 1%). Total fiber count, fiber type percentages, mean fiber cross-sectional area (CSA), as well as fiber CSA of the different fiber types were determined with an automated software analysis program (Image Pro^®^ analyzer software, Media Cybernetics Inc., Rockville, USA).

### Untargeted metabolic profiling

The nitrogen frozen muscle samples from the M. vastus lateralis and the M. pectoralis were shipped on dry-ice to Metabolon Inc. (Durham, NC) for untargeted metabolomic profiling using Ultra High Performance Liquid chromatography/Mass Spectrometry/Mass Spectrometry (UHPLC/MS/MS) and Gas chromatography/ Mass Spectrometry (GC/MS) as previously described [86, 87]. The two columns that were used were a C18 column (Waters UPLC BEH C18-2.1x100 mm, 1.7 μm) and a hydrophilic interaction liquid chromatography (HILIC) column (Waters UPLC BEH Amide 2.1x150 mm, 1.7 μm). The extracts were divided into five fractions: two for analysis by two separate reverse phase (RP)/UPLC-MS/MS methods with positive ion mode electrospray ionization (ESI), one for analysis by RP/UPLC-MS/MS with negative ion mode ESI, one for analysis by Hydrophilic Interaction Ultra Performance Liquid Chromatography/Mass Spectrometry/Mass Spectrometry (HILIC/UPLC-MS/MS) with negative ion mode ESI and one sample was reserved as a backup. All methods utilized a Waters ACQUITY ultra-performance liquid chromatography (UPLC) and a Thermo Scientific Q-Exactive high resolution/accurate mass spectrometer interfaced with a heated electrospray ionization (HESI-II) source and Orbitrap mass analyzer operated at 35,000 mass resolution. In total, 36 samples were analyzed and were run in the same batch. Raw data was then extracted, peaks were identified and quality controls (QC) were processed using Metabolon’s hardware and software. Compounds were identified by comparison to Metabolon’s library entries that contain >3300 purified standard compounds. A detailed overview of the analytic procedures is provided in S1 File.

### Statistical analysis

#### Heart rate follow-up

Data were analyzed using Statistical Analysis System (SAS) version 9.4 for Windows (SAS Institute Inc., Cary, NC). To study the difference in effect of AT versus DT on heart rate parameters a two-sample *t*-test was used. The effect of 8 weeks of DT and AT on heart rate before and after a training session were analyzed using a paired *t*-test. Significance was set at p<0.05.

#### Muscle morphometrics

Data were analyzed using the Statistical Analysis System (SAS) version 9.4 for Windows (SAS Institute Inc., Cary, NC). The effect of either DT or AT on muscle morphometrics was analyzed using a mixed effects model with horse as a random effect and muscle, body side, location, period and their interactions as fixed effects. Since the interaction between muscle and period was significant, separate mixed effects models with horse as random effect and body side, location, period and their interactions as fixed effects were fitted for each muscle. Non-significant interactions were removed from the model. Significance was set at p<0.05.

#### Muscle fiber typing

Different fiber types were counted and classified per type and relative percentages of each type were calculated. Mean CSA was calculated dividing 1 mm^2^ by number of fibers (in μm^2^). Furthermore, fiber CSA of all types of fibers separately was determined (in μm^2^).

Statistical analysis was performed in R (R Core Team, 2019). The results are given as median (minimum-maximum). Significance was set at p<0.05. To compare mean CSA, percentages of fiber types and CSA of each fiber type between muscles, a Kruskal-Wallis test was performed. If this effect was significant, pairwise comparisons between muscle types were tested using a Wilcoxon test on significance level 0.017 (Bonferroni correction for multiple comparisons). For each fiber type of the M. pectoralis, M. vastus lateralis and M. semitendinosus, to test the effect of training on the percentage and the fiber CSA, a Wilcoxon signed rank test was performed.

#### Metabolomics analysis

Data were analyzed using R (version 2.14: www.r-project.org). The present dataset comprises a total of 493 compounds of known identity. Following log transformation and imputation of missing values by the minimum observed value for each compound, ANOVA contrasts, a paired t-test and Welch’s two-sample *t*-tests were performed to identify biochemicals that differed significantly before (untrained horses) and after training (after dry treadmill training DT) in the M. pectoralis and M. vastus lateralis of Friesian horses. Significance was set at p<0.05. The false discovery rate (q-value) was used to address the multiple comparisons.

## Results

### Heart rate follow up

Daily routine check-ups for vital signs were uneventful in both trials. The resting heart rate was significantly higher in the AT group versus DT group before the training period of 8 weeks (37.2 ± 1.30 versus 30.6 ± 1.95; p<0.0001) and after the training period of 8 weeks (36.2 ± 3.74 versus 31.4 ± 2.76; p = 0.0121). In addition, when looking at the heart rate measured after a training session, AT sessions significantly increased heart rate more than DT sessions, and this applied to the measurements both before and after the 8 weeks training period (week 0: 41.8 ± 3.19 versus 32 ± 0; p<0.0001; week 8: 37 ± 3.74 versus 30.9 ± 1.95; p = 0.0019).

A significant increase in heart rate was found directly after AT when compared to the resting heart rate in the unconditioned horses (37.2 ± 1.30 before the AT session versus 41.8 ± 3.19 after the AT session; p = 0.0095). After 10 minutes, heart rate went back to resting values again. However, after 8 weeks of training, the increase in heart rate directly after exercise was not significant anymore (36.2 ± 3.49 before the AT session versus 37.0 ± 3.74 after the AT session).

DT did not significantly change the resting heart rate, nor the heart rate after a training session and this applied to the start (week 0) and finish (week 8) of the training trial.

### Longitudinal follow-up of muscle morphometrics

#### Dry treadmill training

**Fig 5:**
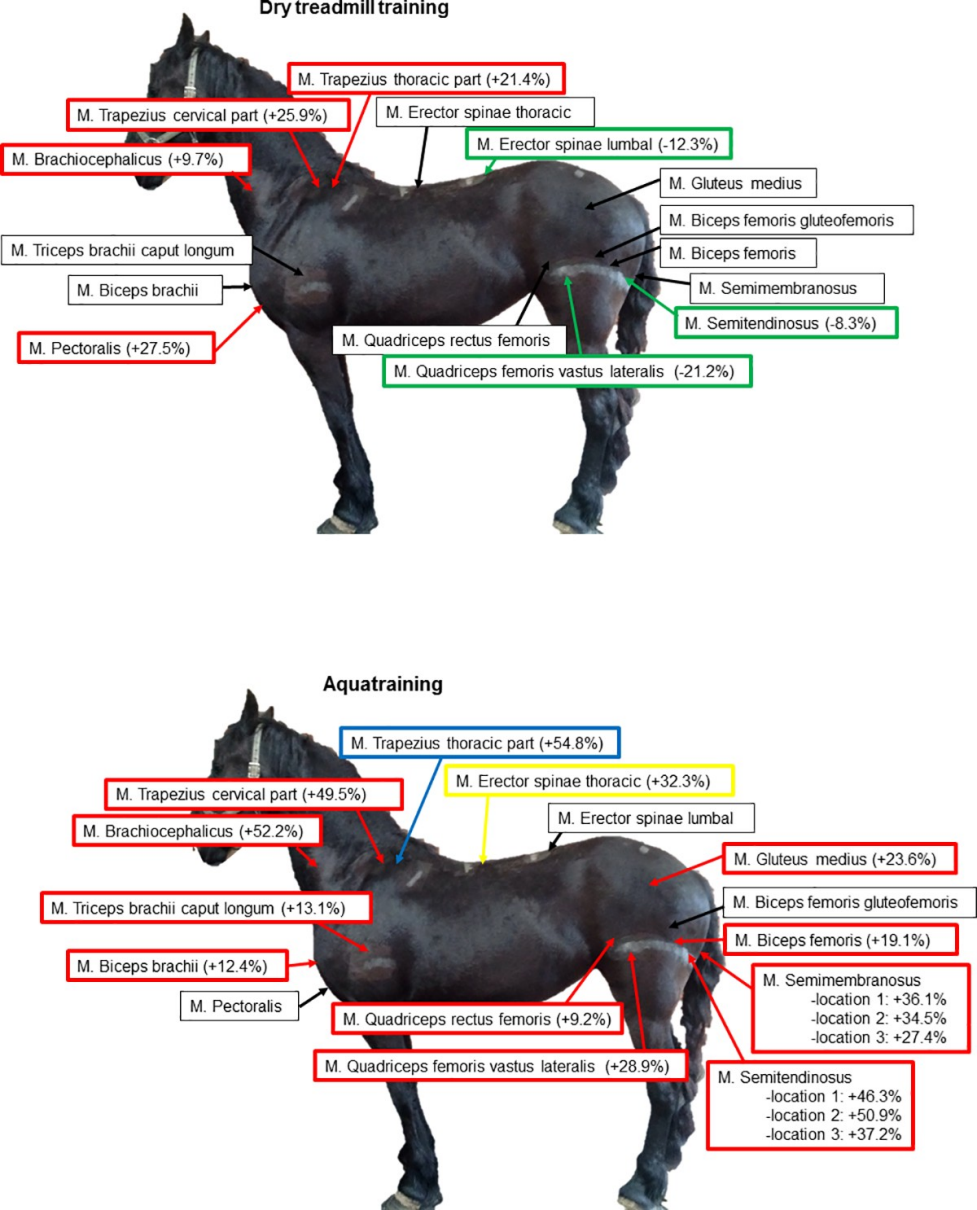
Overview of changes in muscle diameter after 8 weeks of dry treadmill training and 8 weeks of aquatraining. Green: significant decrease in muscle diameter (p<0.05); Red: significant increase in muscle diameter (p<0.05); Yellow: significant increase in muscle diameter right side > left side (p<0.05); Blue: significant increase in muscle diameter left side > right side (p<0.05). (A) Dry treadmill training; (B) Aquatraining.

Predominantly muscles of the forehand increased in muscle diameter. Muscle groups of the hindquarters showed a decrease in muscle diameter. For a clear overview, see Fig 5 and Table 1. The maximal effect of training, which is expressed by either increase or decrease in muscle diameter, was already reached after 4 weeks of training in 7 of the monitored muscles.

**Table 1:** Results of the muscle morphometric study. Evolution of muscle diameter after dry treadmill training and aquatraining.

		Dry treadmill training						Aquatraining					
		Muscle diameter (cm)			Evolution of muscle diameter between time points (p value)			Muscle diameter (cm)			Evolution of muscle diameter between time points (p value)		
	Muscle	start	week4	week8	start to week 4	start to week 8	week 4 to week 8	start	week4	week8	start to week 4	start to week 8	week 4 to week 8
**muscles of the forehand**	trapezius cervical part	1.71	2.23	2.15	**p<0.0001**	**p<0.0001**	p = 0.4957	1.61	2.11	2.41	**p<0.0001**	**p<0.0001**	**p = 0.004**
	brachiocephalicus	1.99	2.37	2.19	**p<0.0001**	**p = 0.005**	**p = 0.0077**	1.71	2.22	2.61	**p<0.0001**	**p<0.0001**	**p = 0.002**
	biceps brachii	4.81	4.76	4.79	p = 0.8412	p = 0.9714	p = 0.9413	4.45	4.88	5.01	**p = 0.0013**	**p<0.0001**	p = 0.5678
	trapezius thoracic part	1.71	2.25	2.07	**p<0.0001**	**p<0.0001**	p = 0.0761	1.46	1.61	2.00	p = 0.1933	**p = 0.0024**	p = 0.1644
	triceps brachii caput longum	6.04	6.23	6.29	p = 0.406	p = 0.2186	p = 0.9195	5.78	6.46	6.54	**p = 0.0002**	**p<0.0001**	p = 0.8666
	pectoralis profundus	1.61	1.90	2.06	**p = 0.0043**	**p<0.0001**	p = 0.1693	1.58	1.65	1.65	p = 0.725	p = 0.6439	p = 0.931
	erector spinae thoracic part	5.58	5.71	5.78	p = 0.5286	p = 0.2109	p = 0.8114	4.95	6.00	6.18	**p<0.0001**	**p<0.0001**	p = 0.50
**muscles of the hindquarters**	erector spinae lumbal part	6.39	5.83	5.67	**p = 0.0159**	**p = 0.0013**	p = 0.701	5.14	5.23	5.53	p = 0.925	p = 0.0847	p = 0.9673
	rectus femoris	6.73	6.94	6.70	p = 0.5549	p = 0.9783	p = 0.4346	5.10	5.44	5.57	**p = 0.0268**	**p = 0.0014**	p = 0.476
	vastus laterlalis	6.68	5.96	5.27	**p = 0.0079**	**p<0.0001**	**p = 0.0119**	5.91	6.48	7.63	p = 0.0805	**p<0.0001**	**p = 0.0001**
	gluteofemoralis	4.42	4.82	4.25	p = 0.0505	p = 0.5534	**p = 0.0026**	4.81	4.01	4.57	**p = 0.0007**	p = 0.4934	**p = 0.0234**
	biceps femoris	10.30	10.19	10.42	p = 0.9091	p = 0.8915	p = 0.6586	8.38	9.55	9.98	**p<0.0001**	**p<0.0001**	p = 0.2059
	semitendinosus	7.19	6.66	6.60	**p = 0.0035**	**p = 0.0008**	p = 0.9134	6.81	7.90	9.67	p = 0.0607	**p<0.0001**	**p = 0.0012**
	semimembranosus	7.61	7.79	7.84	p = 0.4441	p = 0.2598	p = 0.9331	8.23	9.42	10.73	p = 0.1639	**p<0.0001**	**p = 0.0038**
	gluteus medius	5.26	4.66	4.99	**p = 0.0007**	p = 0.1967	p = 0.1036	4.24	4.76	5.24	p = 0.375	**p = 0.0024**	p = 0.176

The muscle diameters are given in cm and were measured at start of the study and after 4 and 8 weeks of dry treadmill training (n = 7) and at start and after 4 and 8 weeks of aquatraining (n = 5). The evolution of muscle diameter between timepoints was compared (from the start of the study to 4 and 8 weeks of training and from week 4 to week 8 of training) and p values for each period are given and marked in red, for the muscles that significantly increased in muscle diameter in that specific period; in green, for the muscles that significantly decreased.

#### Aquatraining

Predominantly muscles of the hindquarters increased in muscle diameter and also several muscle groups of the forehand showed a significant increase in muscle diameter. Also for this training type, the maximal effect was reached already after 4 weeks of training in 6 of the monitored muscles. For a clear overview, see Fig 5 and Table 1. Interestingly, the triangular shaped muscles semitendinosus and semimembranosus showed an asymmetric increase of muscle diameter depending on the measured location (see Fig 3 for an overview of the measured locations) and in both muscles, location 3 (depth at the most distal part of the muscle, see Fig 3B) showed the smallest increase.

### Muscle fiber type composition of different muscle groups and shifts in response to 8 weeks of dry treadmill training

#### Differences in muscle fiber type composition, mean CSA and fiber CSA of different muscle groups in untrained Friesian horses

**Fig 6:**
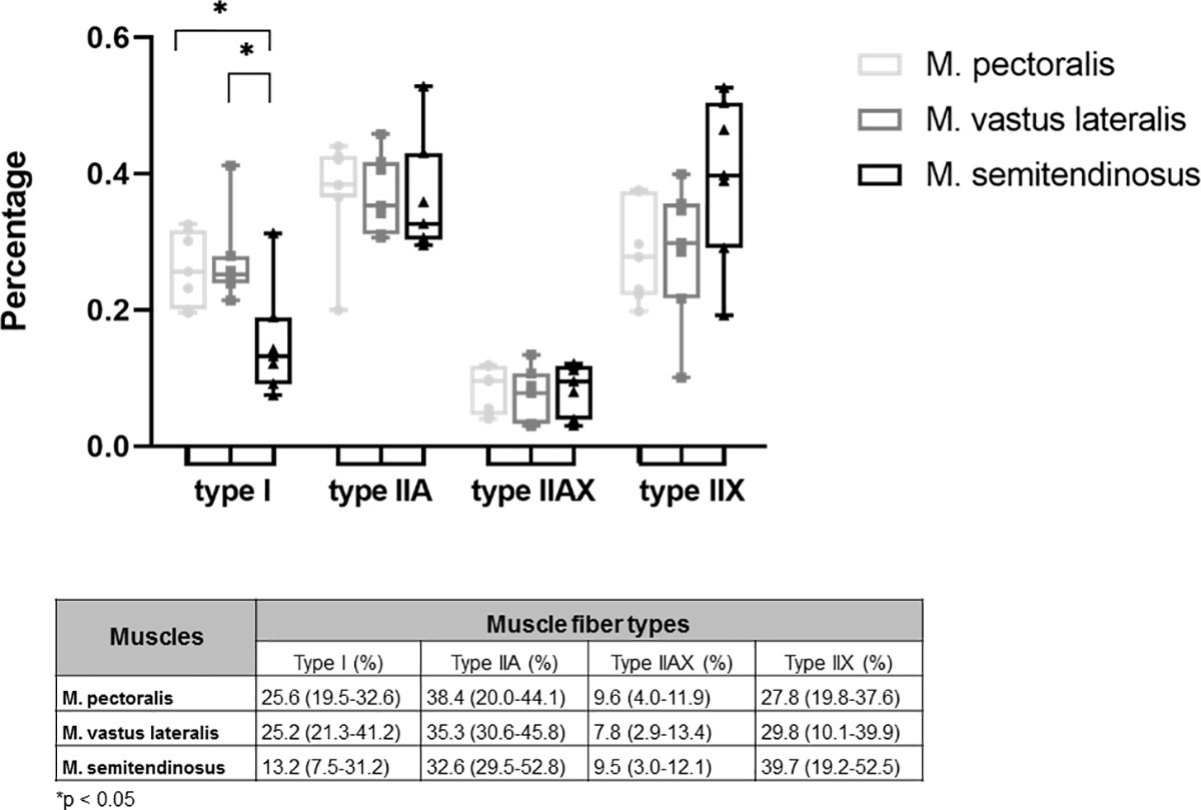
Muscle fiber type composition of M. pectoralis, M. vastus lateralis and M. semitendinosus of untrained Friesian horses. Results are percentages and are given as median (minimum-maximum).

Both M. pectoralis and M. vastus lateralis have a similar composition, whereas the M. semitendinosus contained significantly less type I fibers when compared to the M. pectoralis (p = 0.0111) and the M. vastus lateralis (p = 0.0174). Both M. pectoralis and M. vastus lateralis contained almost 75% fast twitch fibers (Fig 6).

Mean CSA of M. pectoralis was significantly larger than that of the M. vastus lateralis (respectively 6024 μm^2^ (range: 4524–7894 μm^2^) and 3644 μm^2^ (range: 3225–4528 μm^2^) p = 0.0011), but was not significantly different from the M. semitendinosus. The mean CSA of M. semitendinosus was also significantly larger than that of the M. vastus lateralis (respectively 4909 μm^2^ (range: 3644–5907 μm^2^) and 3644 μm^2^ (range: 3225–4528 μm^2^), p = 0.0262).

**Fig 7:**
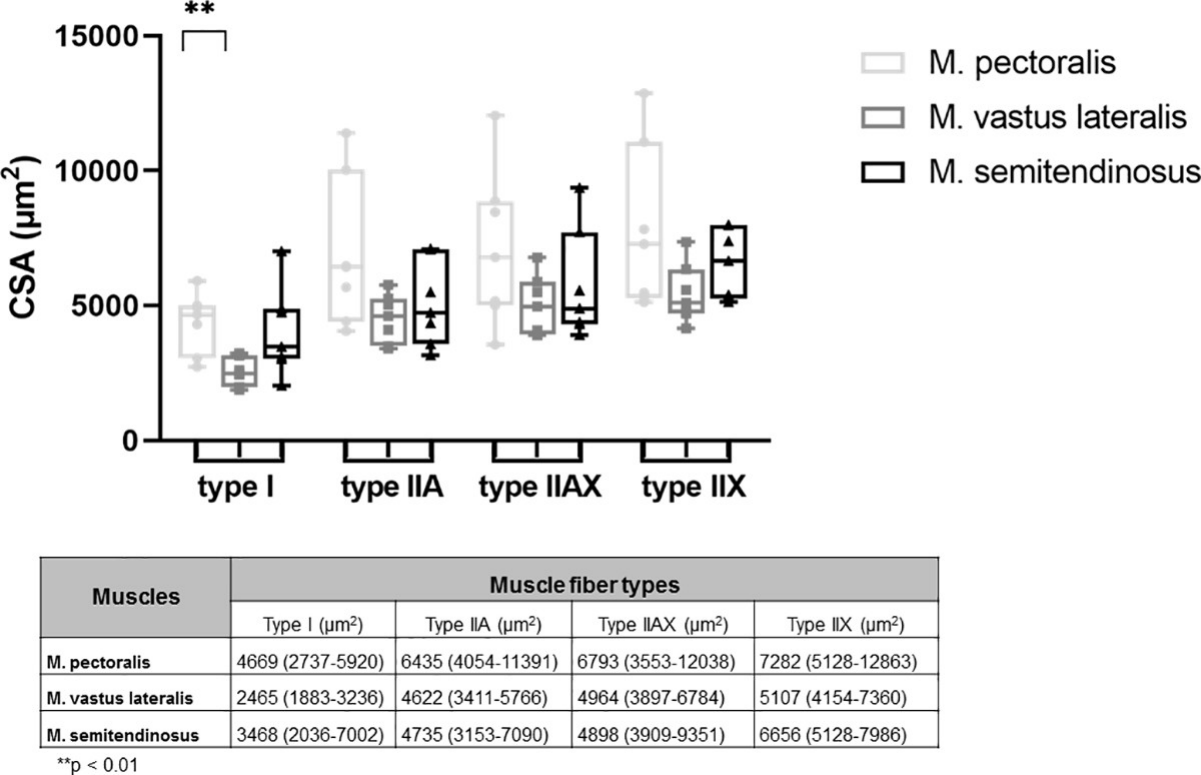
Cross sectional area of type I, IIA, IIAX and type IIX muscle fibers in different muscles. Results are given as median (minimum-maximum) in μm^2^.

When looking at fiber CSA of the different fiber types, these were quite similar for M. pectoralis and M. semitendinosus (Fig 7), however fiber CSA of type I fibers was significantly larger in the M. pectoralis than in the M. vastus lateralis (respectively 4670 μm^2^ (range: 2737–5920 μm^2^) versus 2465 μm^2^ (range: 1883–3236 μm^2^); p = 0.0069).

#### Shifts in muscle fiber type composition, mean CSA and fiber CSA in M. pectoralis, M. vastus lateralis and M. semitendinosus in response to 8 weeks of DT

After 8 weeks of DT, there were significant shifts in muscle fiber type composition in the M. pectoralis and M. vastus lateralis, but not in the M. semitendinosus.

**Fig 8:**
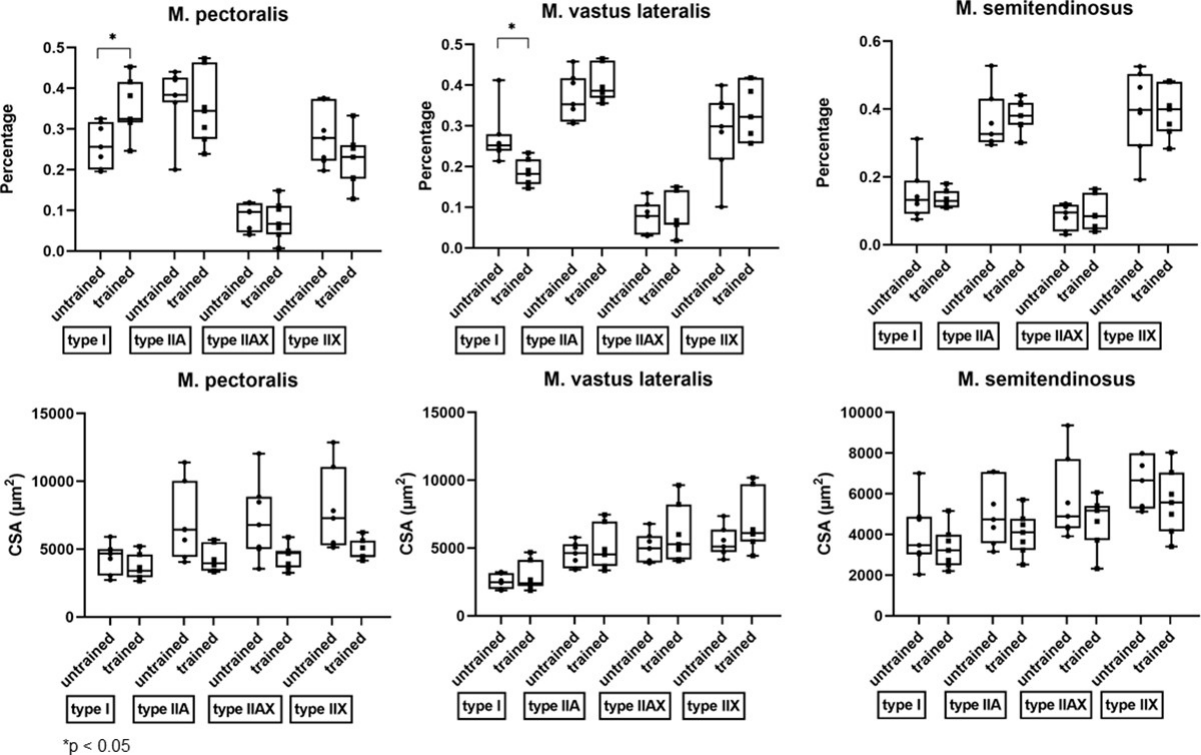
Effect of dry treadmill training on muscle fiber type composition and cross sectional area (CSA). Effect of 8 weeks of DT on muscle fiber type composition and CSA of the different muscle fibers was measured in the M. pectoralis, M. vastus lateralis and M. semitendinosus at rest in unconditioned state (untrained) and after 8 weeks of DT (trained) in Friesian horses.

The M. pectoralis showed a significant increase in expression of type I muscle fibers (p = 0.0156) whereas M. vastus lateralis showed a decreased proportion of type I fibers in response to 8 weeks of DT (p = 0.0153) (Fig 8).

When looking at fiber CSA of each individual fiber type, there was no effect of training on the CSA of the different fiber types (Fig 8).

**Fig 9:**
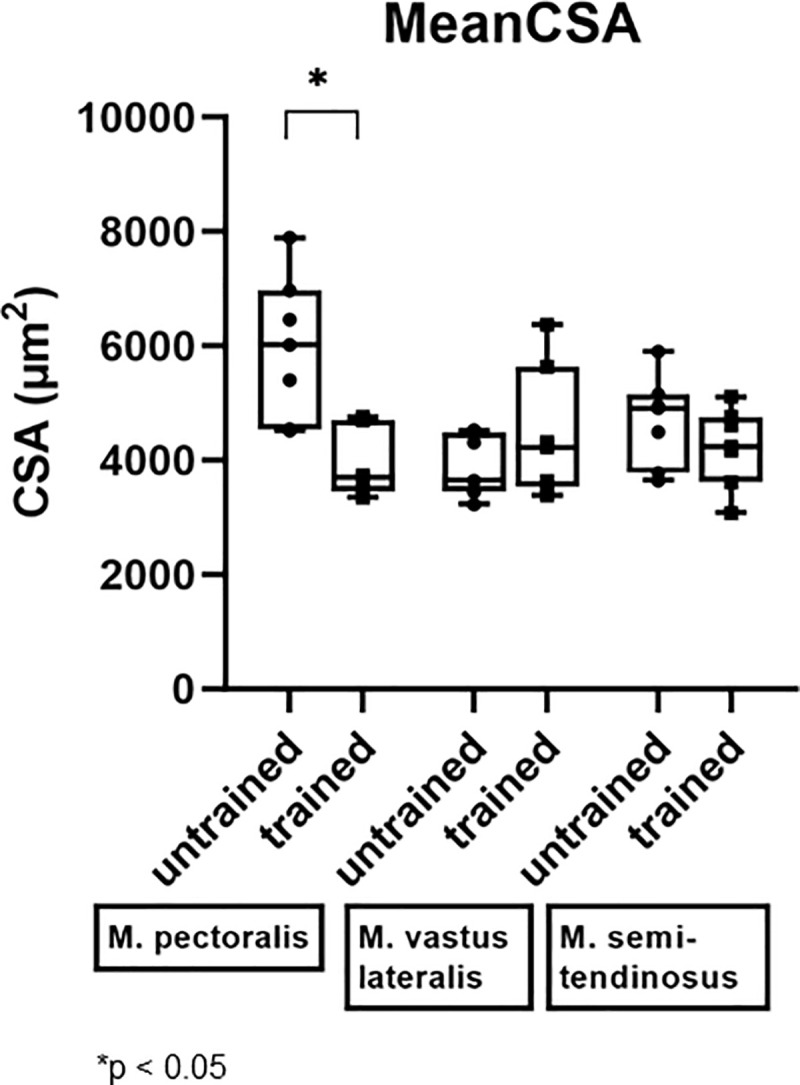
Effect of dry treadmill training on mean cross sectional area (CSA). Effect of 8 weeks of DT on mean CSA was measured in the M. pectoralis, M. vastus lateralis and M. semitendinosus at rest in unconditioned state (untrained) and after 8 weeks of DT (trained) in seven Friesian horses.

A significant decrease in mean CSA of the M. pectoralis, which increased in muscle diameter, was seen after 8 weeks of DT (from 6024 μm^2^ (range: 4524–7895 μm^2^) to 3692 μm^2^ (range: 3349–4761 μm^2^); p = 0.0312) (Fig 9).

### Evolution of muscle metabolomics throughout 8 weeks of DT

**Fig 10:**
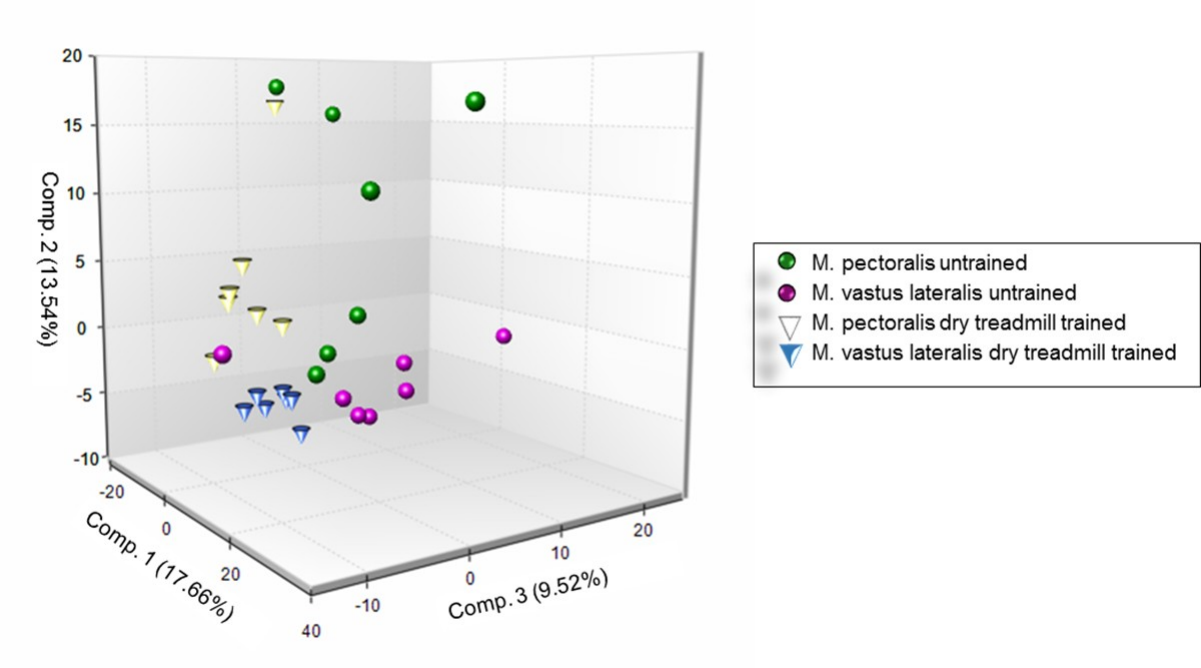
Principal component analysis (PCA) of metabolomic datasets. PCA was performed on the M. pectoralis and the M. vastus lateralis of untrained and dry treadmill trained Friesian horses.

The biochemical profile of 493 different metabolites could be identified. The principal component analysis (PCA) for the detected peaks are shown in Fig 10. Before training, a distinction between both muscle groups can be made. Eight weeks of DT induces a significant shift in metabolic profile of both muscle groups in the same direction. Clustering is much more pronounced after 8 weeks of DT. A significant fold change was detected in respectively 108 metabolites in the M. pectoralis and 114 metabolites in the M. vastus lateralis and 39 metabolites were significantly changed in both muscles in response to exercise, which represents 18% overlap.

#### The fatty acid oxidation pathway is significantly upregulated in predominantly the M. pectoralis in response to 8 weeks of DT

A wide array of β-oxidation pathway intermediates were significantly altered by DT (Table 2: Lipid metabolism, Fig 1). Especially in the M. pectoralis, a significant decrease in long chain fatty acids (0.2- to 0.7-fold) and in polyunsaturated fatty acids (PUFAs) (0.2- to 0.67-fold) could be seen after 8 weeks of DT. This was less pronounced for the M. vastus lateralis. Levels of inflammatory mediators such as n-6 PUFAs (poly unsaturated fats: arachidonate (0.51 fold), linoleate (0.38 fold) and dihomolinoleate (0.40 fold)) and lipid peroxidation (4-hydroxyl-nonenal-gluthatione (0.32-fold) and hydroxy-octadeca-dienoic acids 13-HODE+9-HODE (0.27-fold)) products were significantly decreased in response to 8 weeks of DT in the M. pectoralis. No significant changes in the levels of these inflammatory mediators were detected in the M. vastus lateralis (Table 2: Lipid metabolism).

**Table 2:** Metabolic heatmap of the M. pectoralis and M. vastus lateralis.

		trained (DT) untrained				M. pectoralis M. vastus lateralis	
Sub Pathway	Biochemical Name						
		M. pectoralis	M. vastus lateralis	M. vastus lateralis M. pectoralis	M. pectoralis M. vastus lateralis	Un-trained	DT
Lipid metabolism							
Short Chain Fatty Acid	valerate	1.07	1.14	1.27	0.96	0.90	0.84
	3-hydroxybutyrate (BHBA)	1.18	1.20	1.14	1.24	1.05	1.04
Long Chain Fatty Acid	palmitate (16:0)	**0.60**	0.82	**0.72**	**0.69**	1.14	0.84
	palmitoleate (16:1n7)	**0.37**	0.83	**0.40**	0.77	**2.06**	0.92
	10-heptadecenoate (17:1n7)	**0.48**	0.91	**0.57**	0.77	**1.60**	0.85
	stearate (18:0)	**0.65**	**0.70**	**0.74**	**0.61**	0.94	0.88
	10-nonadecenoate (19:1n9)	**0.45**	1.12	**0.62**	0.81	**1.82**	0.72
	arachidate (20:0)	**0.69**	**0.65**	**0.72**	**0.63**	0.91	0.97
	eicosenoate (20:1)	**0.45**	1.51	0.65	1.05	**2.32**	0.70
	erucate (22:1n9)	0.68	1.07	0.71	1.02	1.50	0.96
	oleate/vaccenate (18:1)	**0.28**	0.84	**0.33**	0.73	**2.56**	0.87
Polyunsaturated Fatty Acid (n3 and n6)	eicosapentaenoate (EPA; 20:5n3)	**0.48**	**0.73**	**0.42**	0.82	**1.72**	1.12
	docosapentaenoate (n3 DPA; 22:5n3)	**0.54**	1.40	0.82	0.92	**1.70**	**0.66**
	docosahexaenoate (DHA; 22:6n3)	0.66	0.77	0.66	0.76	1.15	0.99
	linoleate (18:2n6)	**0.36**	0.84	**0.47**	0.64	**1.77**	0.76
	linolenate [alpha or gamma; (18:3n3 or 6)]	**0.20**	0.65	**0.21**	0.64	**3.13**	0.98
	dihomo-linolenate (20:3n3 or n6)	**0.40**	1.33	**0.55**	0.97	**2.42**	0.73
	arachidonate (20:4n6)	**0.51**	0.95	**0.63**	0.76	**1.50**	0.80
	dihomo-linoleate (20:2n6)	**0.42**	1.53	0.65	0.99	**2.35**	0.65
Fatty Acid Metabolism (also BCAA Metabolism)	butyrylcarnitine (C4)	**0.67**	**0.61**	0.77	**0.53**	0.80	0.87
	propionylcarnitine (C3)	0.85	**0.78**	**0.83**	**0.80**	0.94	1.02
Fatty Acid Metabolism(Acyl Carnitine)	acetylcarnitine (C2)	1.04	0.94	1.02	0.95	0.92	1.01
	3-hydroxybutyrylcarnitine	**1.98**	**1.59**	**1.28**	**2.45**	1.24	**1.54**
	hexanoylcarnitine (C6)	**0.51**	**0.54**	**0.44**	0.62	1.23	1.16
	octanoylcarnitine (C8)	**0.57**	**0.61**	**0.48**	0.72	1.25	1.18
	decanoylcarnitine (C10)	**0.72**	**0.70**	**0.56**	0.91	1.25	1.30
	cis-4-decenoylcarnitine (C10:1)	1.10	1.08	**0.64**	**1.86**	**1.69**	**1.72**
	laurylcarnitine (C12)	**0.64**	**0.50**	**0.55**	**0.58**	0.90	1.16
	myristoylcarnitine (C14)	0.81	**0.74**	**0.49**	1.22	**1.51**	**1.65**
	palmitoylcarnitine (C16)	**0.59**	0.75	**0.56**	0.79	1.35	1.05
	palmitoleoylcarnitine (C16:1)*	1.06	1.22	**0.66**	**1.96**	**1.85**	**1.61**
	stearoylcarnitine (C18)	1.07	0.99	**0.74**	**1.42**	1.32	**1.44**
	linoleoylcarnitine (C18:2)*	**1.42**	**1.42**	0.89	**2.26**	**1.59**	**1.59**
	linolenoylcarnitine (C18:3)*	1.23	**1.42**	0.75	**2.34**	**1.89**	**1.65**
	oleoylcarnitine (C18:1)	1.18	**1.40**	0.81	**2.03**	**1.72**	**1.45**
	myristoleoylcarnitine (C14:1)*	1.09	0.99	**0.59**	**1.81**	**1.66**	**1.83**
	adipoylcarnitine (C6-DC)	**1.54**	**1.41**	**1.62**	**1.34**	0.87	0.95
	arachidoylcarnitine (C20)*	**1.35**	**1.26**	1.02	**1.68**	1.24	**1.33**
	arachidonoylcarnitine (C20:4)	**1.64**	**1.85**	1.04	**2.92**	**1.78**	**1.58**
	adrenoylcarnitine (C22:4)*	**1.75**	1.23	**0.62**	**3.47**	**1.99**	**2.83**
	dihomo-linolenoylcarnitine (20:3n3 or 6)*	1.49	**1.64**	0.79	**3.10**	**2.09**	**1.89**
	dihomo-linoleoylcarnitine (C20:2)*	**1.58**	1.42	0.72	**3.11**	**1.97**	**2.19**
	eicosenoylcarnitine (C20:1)*	**1.55**	**1.37**	**0.65**	**3.24**	**2.09**	**2.37**
	erucoylcarnitine (C22:1)*	1.41	1.18	**0.65**	**2.54**	**1.81**	**2.16**
	docosatrienoylcarnitine (C22:3)*	**2.21**	1.14	**0.59**	**4.30**	**1.94**	**3.77**
	docosapentaenoylcarnitine (C22:5n3)*	**2.02**	1.35	0.76	**3.60**	**1.78**	**2.66**
	docosahexaenoylcarnitine (C22:6)*	**1.84**	1.16	0.77	**2.80**	**1.52**	**2.41**
	margaroylcarnitine*	1.13	1.17	0.83	**1.59**	**1.41**	**1.36**
	pentadecanoylcarnitine (C15)*	1.00	0.90	0.83	1.09	1.09	1.21
Carnitine Metabolism	deoxycarnitine	1.00	1.05	**1.40**	**0.74**	**0.75**	**0.71**
	carnitine	**1.07**	1.04	**1.09**	1.02	0.95	0.98
Glycolysis, Gluconeogenesis, and Pyruvate Metabolism	glucose	1.15	**2.42**	**2.12**	1.31	1.14	**0.54**
	glucose 6-phosphate	1.67	**1.76**	**2.51**	1.17	0.70	0.66
	fructose-6-phosphate	1.38	**1.70**	**3.16**	0.74	**0.54**	**0.44**
	Isobar: fructose 1,6-diphosphate, glucose 1,6-diphosphate, myo-inositol 1,4 or 1,3-diphosphate	1.10	**0.54**	0.69	0.87	0.79	**1.60**
	dihydroxyacetone phosphate (DHAP)	1.00	1.24	1.38	0.90	0.90	0.73
	3-phosphoglycerate	0.62	**0.11**	0.49	**0.14**	**0.23**	1.28
	phosphoenolpyruvate (PEP)	0.47	**0.07**	**0.46**	**0.07**	**0.14**	1.02
	pyruvate	1.08	**3.01**	**2.73**	1.20	1.10	**0.40**
	lactate	1.28	**2.73**	**2.42**	**1.44**	1.13	**0.53**
	glycerate	0.85	**0.52**	1.06	**0.41**	**0.49**	0.80
Fructose, Mannose and Galactose Metabolism	fructose	**1.81**	1.30	1.22	**1.93**	1.07	1.49
	mannitol/sorbitol	0.82	0.98	1.09	0.74	0.90	0.76
	mannose	1.17	**1.91**	**1.47**	**1.53**	1.30	0.80
	mannose-6-phosphate	1.41	**2.39**	**2.94**	1.14	0.81	**0.48**
	galactitol (dulcitol)	**1.51**	1.23	**1.45**	1.28	0.85	1.04
Pentose Phosphate Pathway	6-phosphogluconate	1.18	**1.47**	1.22	**1.43**	1.21	0.97
	ribose 5-phosphate	1.24	**1.32**	**1.54**	1.07	0.86	0.81
	ribose 1-phosphate	**0.59**	0.67	**0.56**	0.71	1.19	1.05
	ribulose/xylulose 5-phosphate	1.42	**4.00**	1.49	**3.82**	**2.69**	0.96
Glycogen Metabolism Pathway	maltotetraose	1.02	1.45	**1.63**	0.91	0.89	**0.62**
	maltotriose	1.07	**1.88**	**2.06**	0.97	0.91	**0.52**
	maltose	0.97	**2.16**	**2.37**	0.88	0.91	**0.41**
TCA Cycle	citrate	1.24	0.92	**0.68**	**1.68**	**1.36**	**1.82**
	aconitate [cis or trans]	1.20	0.97	0.73	**1.60**	1.33	**1.65**
	alpha-ketoglutarate	1.70	**1.86**	1.01	**3.11**	1.83	1.67
	succinylcarnitine (C4-DC)	**1.40**	**1.43**	**1.22**	**1.64**	1.17	1.15
	succinate	**0.69**	0.92	**0.60**	1.06	**1.54**	1.15
	fumarate	1.11	1.19	0.96	**1.39**	1.25	1.16
	malate	1.08	**1.23**	1.15	1.15	1.07	0.94
	2-methylcitrate/homocitrate	**1.77**	**2.26**	1.21	**3.32**	**1.87**	**1.47**
Oxidative Phosphorylation	acetylphosphate	0.98	**0.40**	**0.74**	**0.53**	**0.54**	**1.33**
	phosphate	1.01	**1.56**	**1.48**	1.07	1.05	**0.68**
Proteinogenic BCAA’s isoleucine, leucine and valine metabolism	leucine	0.99	0.98	1.02	0.96	0.96	0.97
	N-acetylleucine	1.39	1.03	1.01	1.42	1.02	1.37
	4-methyl-2-oxopentanoate	0.93	1.03	0.91	1.05	1.13	1.02
	alpha-hydroxyisocaproate	1.00	1.00	1.07	0.93	0.94	0.93
	isovalerylglycine	**1.74**	1.03	1.17	**1.53**	0.88	**1.49**
	isovalerylcarnitine (C5)	**0.46**	**0.67**	0.83	**0.37**	0.81	**0.56**
	beta-hydroxyisovalerate	0.79	1.18	0.84	1.11	1.40	0.94
	beta-hydroxyisovaleroylcarnitine	0.97	0.87	**1.25**	**0.68**	**0.70**	**0.78**
	3-methylglutarylcarnitine (2)	1.12	0.95	1.07	0.99	0.89	1.04
	isoleucine	1.01	1.08	**1.10**	1.00	0.98	0.92
	N-acetylisoleucine	0.81	1.13	0.88	1.04	1.28	0.92
	3-methyl-2-oxovalerate	1.11	1.15	0.94	1.36	1.22	1.19
	alpha-hydroxyisovalerate	1.19	1.19	1.20	1.17	0.99	0.99
	2-methylbutyrylcarnitine (C5)	**0.75**	0.79	0.86	**0.69**	0.92	0.87
	tiglylcarnitine (C5:1-DC)	**1.40**	0.99	**0.59**	**2.37**	**1.69**	**2.38**
	ethylmalonate	1.11	1.17	0.97	**1.34**	**1.21**	1.15
	methylsuccinate	1.02	1.09	0.94	1.18	1.16	1.08
	valine	1.11	**1.18**	**1.26**	1.04	0.94	0.88
	3-methyl-2-oxobutyrate	1.14	1.25	1.14	1.24	1.09	0.99
	2-hydroxy-3-methylvalerate	1.15	1.03	1.32	0.90	0.78	0.87
	isobutyrylcarnitine (C4)	**0.79**	**0.67**	**0.51**	1.04	**1.32**	**1.56**
	isobutyrylglycine	0.76	0.80	0.75	0.81	1.07	1.02
	3-hydroxyisobutyrate	1.12	**1.40**	1.10	**1.42**	**1.27**	1.02
	glycylleucine	1.27	**1.88**	**1.77**	1.34	1.06	**0.71**
	glycylvaline	**1.29**	**2.20**	**2.32**	1.23	0.95	**0.56**
	leucylglycine	1.11	**1.58**	**1.50**	1.17	1.05	**0.74**
	phenylalanylalanine	**1.99**	0.74	**1.66**	0.90	**0.45**	1.20
	prolylglycine	1.11	**1.26**	**1.23**	1.14	1.03	0.91
	valylleucine	1.20	**1.63**	**1.32**	**1.49**	1.24	0.91
Aromatic amino acids (AAA): tryptophan, tyrosine, phenylalanine and histidine metabolism	phenylalanine	1.09	1.10	**1.14**	1.05	0.96	0.96
	N-acetylphenylalanine	0.85	0.94	0.88	0.91	1.07	0.97
	phenyllactate (PLA)	0.98	0.86	0.79	1.06	1.08	1.23
	tyrosine	1.08	**1.23**	**1.19**	**1.12**	1.04	0.91
	4-hydroxyphenylpyruvate	1.61	1.01	0.77	**2.09**	1.30	**2.07**
	3-(4-hydroxyphenyl)lactate	1.27	0.98	**0.76**	**1.64**	1.29	**1.67**
	phenol sulfate	0.82	0.87	**0.68**	1.05	1.28	1.21
	3-methoxytyrosine	**1.81**	**1.78**	**1.92**	**1.68**	0.93	0.94
	O-methyltyrosine	1.08	1.13	**1.34**	0.91	0.84	**0.81**
	p-cresol-glucuronide*	**2.69**	**2.91**	**2.12**	**3.68**	1.37	1.27
	3-hydroxyphenylacetatoylcarnitine	1.31	**1.55**	**3.89**	**0.52**	**0.40**	**0.34**
	tryptophan	**1.19**	**1.24**	**1.23**	**1.19**	1.00	0.96
	indolepropionate	1.19	1.19	1.00	1.41	1.19	1.18
	3-indoxyl sulfate	1.23	1.32	1.15	1.41	1.14	1.07
	indolelactate	1.15	1.02	0.84	1.39	1.21	1.37
	kynurenine	**1.16**	1.08	1.05	**1.19**	1.03	1.10
	kynurenate	0.84	1.16	0.75	1.28	1.53	1.11
	N-formylanthranilic acid	0.65	1.45	0.83	1.14	**1.76**	0.79
	tryptophan betaine	**0.52**	**0.39**	**0.51**	**0.39**	0.76	1.02
	C-glycosyltryptophan	0.99	1.10	**0.79**	**1.38**	**1.40**	**1.24**
	histidine	0.97	0.90	**0.75**	**1.16**	**1.19**	**1.29**
	1-methylhistidine	1.14	1.02	**1.30**	0.89	**0.78**	0.87
	trans-urocanate	1.39	2.14	**2.34**	1.28	0.91	0.59
	cis-urocanate	**2.63**	**4.75**	**5.65**	2.21	0.84	0.47
	imidazole lactate	1.07	0.87	**0.71**	**1.32**	**1.23**	**1.51**
	carnosine	**1.11**	**1.10**	**1.17**	1.05	0.94	0.95
	histamine	0.70	0.93	**0.36**	**1.80**	**2.58**	**1.93**
	1-methylhistamine	1.11	1.14	0.78	**1.64**	1.47	1.43
	1-methylimidazoleacetate	0.94	0.82	**0.70**	1.10	1.17	**1.35**
	histidine methyl ester	**1.53**	1.43	1.40	**1.55**	1.02	1.09
Dipeptide Derivative	N-acetylcarnosine	1.25	0.98	0.84	**1.46**	1.17	**1.48**
	homocarnosine	**1.44**	**1.40**	**2.43**	**0.83**	**0.58**	**0.59**
	anserine	**1.18**	0.93	1.01	1.09	0.92	**1.17**
Glutamate Metabolism	glutamate	**1.19**	0.98	1.12	1.05	0.88	1.06
	glutamine	**1.46**	1.00	**2.06**	**0.71**	**0.49**	**0.71**
	N-acetylglutamate	1.32	1.06	1.16	1.22	0.92	1.14
	N-acetylglutamine	**1.54**	0.94	**1.49**	0.97	**0.63**	1.03
	glutamate, gamma-methyl ester	**1.33**	0.99	0.91	**1.44**	1.08	**1.46**
	pyroglutamine*	**1.45**	**1.21**	**1.66**	1.06	**0.73**	0.87
	N-acetyl-aspartyl-glutamate (NAAG)	**1.81**	0.81	1.04	1.39	0.77	**1.73**
	beta-citrylglutamate	0.93	0.81	**0.49**	**1.53**	**1.65**	**1.88**
Glycine, Serine and Threonine Metabolism	glycine	**0.79**	1.01	**1.23**	**0.64**	**0.82**	**0.64**
	N-acetylglycine	**0.78**	**0.84**	**0.70**	0.93	**1.20**	1.11
	sarcosine	**2.22**	**3.10**	**3.14**	**2.19**	0.99	**0.71**
	dimethylglycine	**1.49**	**1.53**	**1.86**	**1.23**	**0.82**	**0.80**
	betaine	**1.49**	**1.47**	**1.53**	**1.43**	0.96	0.98
	serine	**1.45**	**1.89**	**1.50**	**1.83**	**1.26**	0.97
	N-acetylserine	1.06	**1.31**	1.11	**1.25**	1.18	0.95
	threonine	1.04	1.18	1.04	1.18	1.13	1.00
	N-acetylthreonine	0.97	1.14	**1.20**	0.92	0.95	**0.81**
Alanine and Aspartate Metabolism	alanine	1.07	**1.23**	1.05	**1.26**	**1.17**	1.02
	N-acetylalanine	1.09	1.09	0.91	**1.30**	**1.19**	**1.19**
	N-methylalanine	1.01	1.08	**1.47**	**0.74**	**0.73**	**0.69**
	aspartate	**0.53**	**0.67**	0.74	**0.48**	0.90	0.71
	N-acetylaspartate (NAA)	1.21	0.85	**3.80**	**0.27**	**0.22**	**0.32**
	asparagine	0.93	1.06	0.83	1.20	**1.28**	1.13
	N-acetylasparagine	1.09	**0.80**	0.89	0.98	0.90	**1.22**
Lysine Metabolism	lysine	**0.80**	**0.82**	0.90	**0.73**	0.91	0.89
	N6-acetyllysine	**1.38**	0.94	1.05	**1.23**	0.89	**1.32**
	N6,N6,N6-trimethyllysine	1.20	1.23	**1.70**	0.87	**0.72**	**0.71**
	5-(galactosylhydroxy)-L-lysine	0.99	**1.39**	0.91	**1.51**	**1.53**	1.09
	saccharopine	**0.35**	**0.15**	**0.18**	**0.30**	0.86	**1.97**
	2-aminoadipate	**1.35**	**0.52**	**0.66**	1.07	0.79	**2.05**
	glutarate (pentanedioate)	0.97	0.76	0.91	0.82	0.84	1.07
	glutarylcarnitine (C5-DC)	1.08	**0.52**	**0.62**	0.90	0.83	**1.74**
	pipecolate	1.06	1.09	1.19	0.97	0.92	0.89
	6-oxopiperidine-2-carboxylate	1.08	0.94	1.04	0.98	0.91	1.04
	5-aminovalerate	1.01	0.82	1.15	**0.73**	**0.72**	0.88
Methionine, Cysteine, SAM and Taurine Metabolism	methionine	1.07	**1.13**	1.11	1.09	1.02	0.97
	N-acetylmethionine	1.25	1.26	1.05	**1.50**	1.20	1.19
	N-formylmethionine	**1.31**	1.01	**0.74**	**1.78**	**1.36**	**1.76**
	S-methylmethionine	**1.68**	**1.25**	**1.67**	**1.26**	**0.75**	1.00
	methionine sulfone	**1.54**	**1.29**	**1.92**	1.03	**0.67**	0.80
	methionine sulfoxide	**0.84**	0.96	**0.72**	1.11	**1.33**	1.16
	N-acetylmethionine sulfoxide	0.89	0.95	0.56	1.53	1.71	1.60
	S-adenosylmethionine (SAM)	**1.44**	**1.59**	**2.24**	1.02	**0.71**	**0.64**
	S-adenosylhomocysteine (SAH)	1.03	1.04	0.98	1.10	1.06	1.05
	cysteine	1.13	**1.44**	**1.48**	1.10	0.98	0.76
	S-methylcysteine	**1.52**	1.26	1.28	**1.50**	0.99	1.19
	S-methylcysteine sulfoxide	**1.66**	**1.96**	**2.21**	**1.48**	0.89	0.75
	hypotaurine	1.21	**0.70**	**0.70**	1.22	1.00	**1.73**
	taurine	**1.28**	**0.77**	**0.74**	**1.34**	1.05	**1.73**
	N-acetyltaurine	1.18	**0.41**	**0.26**	**1.87**	**1.59**	**4.57**
	taurocyamine	1.14	0.88	1.03	0.98	0.86	1.11
Arginine, ornithine and Proline Metabolism	arginine	1.06	1.13	**1.33**	0.90	**0.85**	**0.80**
	argininosuccinate	**1.77**	1.18	0.83	**2.50**	**1.42**	**2.13**
	urea	1.09	1.12	**1.21**	1.01	0.93	0.91
	ornithine	**2.02**	**1.33**	**1.66**	**1.62**	0.80	1.22
	2-oxoarginine*	0.90	1.05	1.00	0.94	1.04	0.90
	citrulline	1.09	**1.11**	**1.50**	**0.80**	**0.74**	**0.72**
	homoarginine	**1.78**	**1.53**	**1.95**	**1.39**	**0.78**	0.91
	homocitrulline	0.84	**0.64**	0.86	**0.63**	**0.75**	0.98
	proline	**1.16**	**1.39**	**1.28**	**1.26**	1.08	**0.91**
	dimethylarginine (SDMA + ADMA)	**1.26**	1.04	**1.28**	1.03	**0.81**	0.98
	N-acetylarginine	**1.59**	1.17	**1.52**	**1.23**	**0.77**	1.05
	N-delta-acetylornithine	**1.39**	**1.39**	**1.47**	**1.32**	0.95	0.94
	trans-4-hydroxyproline	0.90	1.01	**0.86**	1.05	**1.17**	1.05
	N-methylproline	**1.31**	1.07	**1.27**	1.09	**0.84**	1.03
	argininate*	1.07	1.05	1.14	0.98	0.92	0.94
Glutathione metabolism	glutathione, reduced (GSH)	**1.60**	**1.57**	**3.33**	0.75	**0.47**	**0.48**
	glutathione, oxidized (GSSG)	**1.74**	0.76	0.94	1.40	0.81	**1.84**
	S-methylglutathione	1.37	0.82	0.94	1.19	0.87	1.45
	S-lactoylglutathione	**2.13**	0.74	**3.00**	**0.52**	**0.25**	0.71
	cysteinylglycine	1.21	**2.24**	**3.47**	0.78	0.65	**0.35**
	5-oxoproline	**1.57**	1.18	**2.40**	**0.77**	**0.49**	**0.65**
	2-hydroxybutyrate/2-hydroxyisobutyrate	1.10	**1.32**	**1.25**	1.17	1.06	0.88
	ophthalmate	1.44	1.14	1.30	1.27	0.88	1.11
	4-hydroxy-nonenal-glutathione	**0.32**	0.86	0.45	0.61	1.91	0.71

Metabolic profile between muscles in untrained condition and after 8 weeks of dry treadmill training (DT) is compared. The different metabolites are listed and their fold change is given and marked in red when significantly increased and green when significantly decreased (p<0.05) and light red and light green when p<0.1. DT/untrained compares the metabolic profile of trained horses with untrained horses in the M. pectoralis and the M. vastus lateralis. The columns DT/untrained for M. vastus/M. pectoralis and for M. pectoralis/ M. vastus represent the integration of the muscle and training effects and is thus a comparison of metabolic profile of trained over untrained (DT/trained) condition and integrates the comparison between M. pectoralis and M. vastus lateralis (M. pectoralis/M. vastus lateralis and M. vastud lateralis/M. pectoralis). Furthermore muscles are compared with each other (M. pectoralis/M. vastus lateralis) in untrained and in trained condition (DT).

No significant changes in short chain fatty acids such as butyrate and valerate could be detected in both muscle groups. In both M. pectoralis and M. vastus lateralis, a significant upregulated activity at the level of long chain acylcarnitine metabolism was seen after 8 weeks of DT (1.35–1.21 fold), whereas a downregulation of short- and medium chain acylcarnitines was found in both muscle groups.

#### The carbohydrate metabolism pathway is significantly upregulated in response to 8 weeks of DT in the M. vastus lateralis, not in the M. pectoralis

No significant fold changes in carbohydrate metabolism activity could be detected in the M. pectoralis after training. On the other hand, in the M. vastus lateralis a clear upregulation of carbohydrate metabolism pathways could be seen (Table 2: Carbohydrate metabolism: Glycolysis, gluconeogenesis and pyruvate metabolism; Glycogen metabolism pathway). A significant increase in glycogen breakdown intermediates such as maltotriose (1.88 fold) and maltose (2.16 fold) in combination with an increase in early-stage glycolytic intermediates such as glucose (2.42 fold), glucose-6-phosphate (1.76 fold), fructose-6-phosphate (1.70 fold) and a decrease in intermediate stage glycolytic intermediates such as (glycerate, 3-phosphoglycerate, PEP and fructose 1,6-diphosphate, respectively 0.52 fold; 0.11 fold; 0.07 fold; 0.54 fold) indicate an upregulation of glycogenolytic and glycolysis pathways (Fig 1). Likewise, lactate (2.73 fold) and pyruvate (3.01 fold) were significantly increased after 8 weeks of DT.

#### TCA cycle was significantly upregulated in the M. pectoralis when compared to the M. vastus lateralis in response to 8 weeks of DT

In the M. pectoralis, in conjunction with the previously mentioned upregulation of fatty acid metabolism, there was a significant upregulation of the TCA cycle, which oxidizes acetyl-CoA derived from the aerobic glycolysis and the β-oxidation (Table 2: TCA cycle, DT/untrained; M. pectoralis/M. vastus lateralis; Fig 1). This upregulation was visible across most TCA cycle metabolites.

### The pentose phosphate pathway (PPP) was significantly upregulated in response to 8 weeks of DT in the M. vastus lateralis

The PPP pathway, which is a metabolic pathway parallel to glycolysis (Fig 1) remained almost unchanged in the M. pectoralis, however, was significantly upregulated in the M. vastus lateralis (Table 2: Carbohydrate metabolism: Pentose phosphate pathway). The intermediates covering the full pathway of the cycle, such as 6-phosphogluconate (1.47 fold), ribose-5-phosphate (1.32 fold) and ribulose/xylulose-5-phosphate (4 fold) were significantly increased in the M. vastus lateralis after 8 weeks of DT.

#### Amino acid metabolism was significantly upregulated in response to 8 weeks of DT

*BCAA metabolism was significantly upregulated in the M*. *vastus lateralis in response to 8 weeks of DT*. No significant changes were detected in the BCAA metabolism (leucine, isoleucine and valine) in the M. pectoralis, however, there was a significant increase in several different BCAA metabolites, more specifically BCAA dipeptides, in the M. vastus lateralis after 8 weeks of DT (Table 2: Amino acid metabolism: Proteinogenic BCAAs): glycylleucine (1.88 fold), glycylvaline (2.20 fold), leucylglycine (1.58 fold) and valylleucine (1.63 fold), indicating increased BCAA anabolism (Fig 2).

*Aromatic amino acid (AAA) metabolism was significantly upregulated in both M*. *pectoralis and M*. *vastus lateralis in response to 8 weeks of DT*. The essential AAAs phenylalanine and histidine showed very little significant changes in response to 8 weeks of DT. Carnosine, a dipeptide derived from histidine and β-alanine, showed a significant upregulation in response to 8 weeks of DT in both M. pectoralis and M. vastus lateralis (respectively 1.44 and 1.40 fold increase).

Tryptophan showed a nearly significant increase in both M. pectoralis (1.19 fold) and a significant increase in the M. vastus lateralis (1.24) in response to 8 weeks of DT. This was associated with a significant decrease in tryptophan betaine (respectively 0.52 and 0.39 fold). Also, tyrosine metabolism was significantly upregulated in both muscles, for example p-cresol-glucuronide (respectively 2.69 and 2.91 fold increase) and 3-methoxythyrosine (respectively 1.81 and 1.78 fold increase) (Table 2: Amino acid metabolism: Aromatic amino acids).

*Glutamine/glutamate metabolism was significantly upregulated in the M*. *pectoralis*, *not in the M*. *vastus lateralis after 8 weeks of DT*. Glutamate is known to be an important metabolic hub for synthesis of various amino acids, nucleic acids, nucleotides and co-factor biosynthesis. Glutamine (1.46 fold) and glutamate (1.19 fold), together with other metabolites of the glutamine/glutamate metabolism (i.e. N-acetylglutamine, N-acetyl-aspartyl-glutamate (NAAG)), were significantly upregulated in the M. pectoralis after 8 weeks of DT (Table 2, Amino acid metabolism: Glutamate metabolism; Fig 1).

*Glycine and serine metabolism were significantly upregulated in response to DT in both M*. *pectoralis and M*. *vastus lateralis*. Glycine metabolism is importantly involved in production of specialized molecules such as heme, purines and creatine and it is a key building block of collagen. Both glycine (0.79 fold) and acetyl-glycine (0.78 fold) were significantly decreased in the M. pectoralis after 8 weeks of DT, whereas a significant increase in intermediates of glycine metabolism, including sarcosine, betaine and serine was seen in both muscle groups after 8 weeks of DT (2.22 fold, 1.45 fold and 1.49 fold in the M. pectoralis respectively and 3.10 fold, 1.47 fold, 1.89 fold in the M. vastus lateralis) (Table 2, Amino acid metabolism: Glycine, serine and threonine metabolism).

*The cysteine*, *methionine and taurine metabolism showed differential changes in response to 8 weeks of DT*. Cysteine is a non-essential amino acid that is required for protein synthesis and for synthesis of non-protein compounds such as taurine, co-enzyme A, etc. Methionine is involved in folate metabolism, nucleotide synthesis and control of redox status. Cysteine metabolism intermediates were upregulated in M. pectoralis after 8 weeks of DT and showed a fold increase of 1.52 for S-methylcysteine and 1.66 for S-methylcysteine sulfoxide. In the M. vastus lateralis, cysteine increased 1.44 fold and methylcysteine sulfoxide 1.96 fold.

Methionine metabolism was upregulated in both M. pectoralis and M. vastus lateralis. Intermediates of methionine metabolism S-methylmethionine increased respectively 1.68 and 1.25 fold, methionine sulfone 1.54 and 1.29 fold and S-adenosylmethionine (SAM) a 1.44 and 1.59 fold. Methionine was unchanged in the M. pectoralis but increased 1.13 fold in the M. vastus lateralis.

Taurine was significantly increased in the M. pectoralis (1.28 fold) and significantly decreased in the M. vastus lateralis (0.77 fold) and hypotaurine and N-acetyltaurine, which remained unchanged in the M. pectoralis, decreased significantly in the M. vastus lateralis (respectively 0.77 and 0.41 fold) (Table 2: Amino acid metabolism: Methionine, cysteine, SAM and taurine metabolism).

*Proline and arginine metabolism were significantly upregulated*, *especially in M*. *pectoralis*, *after 8 weeks of DT*. Ornithine increased in the M. pectoralis and M. vastus lateralis, respectively 2.02 and 1.33 fold; citrulline was unchanged in the M. pectoralis and increased 1.11 fold in M. vastus lateralis; arginosuccinate increased 1.77 fold in the M. pectoralis and remained unchanged in the M. vastus lateralis. In the M. pectoralis, intermediates of arginine and ornithine metabolism increased significantly in response to 8 weeks of DT: respectively homoarginine 1.78 fold, dimethylarginine 1.26, N-acetylarginine 1.59 and N-delta acetylornithine 1.39 fold increase. The intermediate homoarginine increased a 1.53 in the M. vastus lateralis, whereas the intermediate homocitrulline decreased a 0.64 fold (Table 2: Amino acid metabolism: Arginine, ornithine and proline metabolism).

Proline increased significantly in both muscles in response to DT (1.16 fold in M. pectoralis and 1.39 fold in M. vastus lateralis) and N-methylproline increased in M. pectoralis (1.31 fold) but not in M. vastus lateralis.

#### Glutathione metabolism was altered in both muscle groups in response to 8 weeks of DT

Interestingly, levels of oxidized glutathione (GSSG) were significantly increased in M. pectoralis (1.74 fold) in response to DT, but not in M. vastus lateralis. This was accompanied by an increased level of 5-oxoproline (1.57 fold), a degradation product of GSH. Levels of reduced glutathione (GSH) tended to increase in both muscle groups after 8 weeks of DT (respectively 1.60 in M. pectoralis and 1.57 fold in M. vastus lateralis). At last, it is clear that DT impacted the M. vastus lateralis more than the M. pectoralis, since almost all intermediates of glutathione metabolism increased significantly when comparing trained (DT)/untrained M. vastus lateralis over M. pectoralis (Table 2: Amino acid metabolism; Glutathione metabolism).

## Discussion

Training of horses is still done quite empirically, which is not always favorable for both the horse and the horse owner. This is the first study to apply a standardized multi-modal approach combining longitudinal follow-up of muscle diameter, muscle fiber type composition and untargeted muscle metabolomics in a set of strategically chosen muscles. The study creates a reference baseline for future training studies, working towards the creation of optimally efficient, effective and breed specific and discipline-specific training programs. Thanks to the multi-modal approach a first glimpse is obtained on the interaction between training, muscle plasticity and training-induced shifts in muscle metabolism. It was chosen to apply untargeted metabolomics because it allows for obtaining a thorough 360° view on muscle metabolism in all its diversity and not only focusing on the “well known” energy pathways. This is the first equine study to apply untargeted metabolomics and combining it with longitudinal follow-up of muscle morphometrics and muscle fiber typing.

### Heart rate

AT induced more effects on heart rate compared to DT, and this is in accordance with Greco-Otto et al. (2017) who reported similar findings in Quarter horses. Apparently, AT represents a more important training load, at least with the currently applied training protocol, when compared to DT [80]. This is confirmed by the evolution of a decreased heart rate seen after acute AT sessions after conditioning for 8 weeks. For DT, no effect was seen, neither before or after 8 weeks of training, nor before or after a training session. One of the possible explanations could be the rather low intensity of DT.

### Muscle diameter

When looking at both training techniques, AT has a much more generalized pronounced effect on muscle growth and this is most pronounced for the muscles of the hindquarters, though also muscles of the forehand are influenced. DT on its turn predominantly modulates muscles of the forehand, though to a much lesser extent. From a kinematic point of view, AT induces hypertrophy of muscles involved in elevation and forward movement of the forelimb, flexion of the hind limb and muscles used for creating a more ‘upright’ position [88]. DT, on its turn, modulates forehand muscles involved in abduction, forward movement and suspension of the forelimbs and decreases the diameter of muscles of the hind limbs involved in straightening the hip, knee and hock joint, straightening of the back and flexion of the knee. At first sight, it seems atypical that some muscles of the hind limbs such as M. vastus lateralis and M. semitendinosus decrease in muscle diameter in response to DT, but when looking at mean CSA of these muscles, no significant change is seen after DT. It can thus be concluded that this is not a decrease in muscle diameter *per se*, but rather a relative decrease in muscle diameter most probably due to a reduction of intramuscular adipose tissue depots. Indeed, when measuring muscle diameter with ultrasound, intramuscular fat is also taken into account and thus influences the measured diameter [89]. Application of ultrasound for longitudinal follow-up of muscle diameter obviously has its shortcomings. However, unlike in human, in horses it is practically impossible to apply repetitive CT scan follow-up for this purpose, since it would require general anesthesia on each occasion and on top of that the horses’ core body and legs above knee and elbow do not fit inside the largest bore CT scanners. Several studies have discussed the reducing effect of exercise on intramuscular adipose tissue depots and its enhancing effect on insulin sensitivity in humans, dogs and horses [90–95]. However, none of these studies has additionally involved evolution of muscle fiber CSA. Several studies have reported on the presence of a fair amount of intramuscular fat in equine muscles [96, 97].

#### Maximal effect of training on muscle diameter

In the current study, muscle diameter measurements were performed in the beginning of the study, after 4 and after 8 weeks of training to obtain a better view on when maximal morphometric effects are reached. As mentioned previously, AT had a much more pronounced overall muscle modulating effect when compared to DT and this was seen throughout the entire training period of 8 weeks, during which the intensity and duration of exercise remained unchanged. Maximal growth was reached in 7 and 6 muscle groups in answer to respectively DT and AT after already 4 weeks of training. This is crucial information to set up optimal efficient and cost efficient training protocols, and therefore, it can be suggested that exercise intensity and/or duration should be increased after 4 weeks of this type of exercise.

### Muscle fiber type composition

#### Effect of muscle

Horses are known to contain more fast twitch than slow twitch fibers and this was reflected in all muscle biopsies in this study, with type IIA being identified as predominant fiber type. When looking at muscle fiber type composition, important differences were seen between the three biopsied muscles. The M. pectoralis and M. vastus lateralis are very alike with respect to fiber type composition, however, the M. semitendinosus contains a significant lower amount of type I fibers (15%) when compared to the M. pectoralis and M. vastus lateralis. Physiologically, differences in fiber type composition between muscles can be attributed to differences in their physiological function. It has been shown that postural muscles contain a greater amount of small aerobic slow twitch type I fibers, whereas locomotor muscles are mainly composed of fast twitch either aerobic (type IIA or type IIX) or anaerobic (type IIB) muscle fibers [98–100] and in between these two archetypes there is of course a wide range of distribution options possible depending on the fact whether a certain muscle has a more posture like versus locomotion like function. As mentioned previously, before training, both M. pectoralis and M. vastus lateralis showed a similar muscle fiber type composition. In view of the identified muscle fiber type distributions it can be envisioned that both M. pectoralis and M. vastus lateralis cover besides their predominant locomotor function, also a postural role, which is not the case for the M. semitendinosus. This is supported by Payne et al. (2005) who ascribe an important role to the M. pectoralis in the adduction and stabilization of the forelimb and for the M. vastus lateralis an important role for extension of the stifle. The M. semitendinosus on its turn, has a role in extension of the hip during stance, flexion of the stifle and extension of the hock during swing, which shows that this muscle is pure locomotor and explains why this muscle has a greater amount of fast twitch fibers [101, 102].

The mean CSA provides a view on the average muscle fiber size, across all fiber types. In our study, the mean CSA of the M. pectoralis and M. semitendinosus were similar and were greater than the mean CSA of the M. vastus lateralis. Furthermore, fiber CSA of type I fibers was larger in the M. pectoralis when compared to the M. vastus lateralis. It would be interesting to investigate whether this coincides with a more pronounced basic storage capacity for energy reserves in these muscles. For example, it was shown by a study of Jaworowski et al., (2002) that the activity of lactate dehydrogenase and phosphofructokinase, two important enzymes for the glucose metabolism, were correlated with CSA of type II fibers [103].

#### Effect of training

Training clearly affects both fiber type composition and mean CSA of different muscles. Despite the fact that the M. pectoralis and M. vastus lateralis have comparable muscle fiber type compositions, they show a different shift in fiber type composition in response to the same DT protocol. Type I fibers significantly increased in the M. pectoralis in response to 8 weeks of DT, whereas they significantly decreased in the M. vastus lateralis. This can be explained by the fact that DT challenges these muscle groups in a different way. In general, low intensity exercise will induce shifts in muscle fiber types more from IIX to IIA and from IIA to type I and this has been shown in different species, such as rats [104], mice [105] and horses [38, 106, 107]. Several studies have looked into the effect of treadmill exercise on muscle fiber type composition in horses, but results were equivocal due to differences in applied training protocols, differences in biopsied muscles, age and breed of the enrolled horses. Some studies found no change in muscle fiber type composition [108, 109], whereas other studies did describe shifts in muscle fiber type composition [49, 110]. Hodgson et al. (1985) published a study of 4 horses trained on a treadmill for 7 weeks. They applied training sessions consisting of 1 min at 110 m/min followed by 5 min at 200 m/min. The second training interval was gradually increased each week until 12 min duration. No significant changes in muscle fiber type composition of the M. gluteus medius could be detected, nor were there changes in capillary content of muscle fibers [108]. Likewise, Essén-Gustavsson (1989) reported no change in muscle fiber type composition after 5 weeks of high speed treadmill training in the M. gluteus medius. However, they did detect a significant decrease in the CSA of type IIA fibers and a significant increase in capillary density, which matches with an increase in aerobic capacity [109].

When looking at evolution of mean CSA across muscle fibers, in the current study, it decreased significantly in response to training only in the M. pectoralis. The fiber CSA of the different fiber types remained unchanged with training in all 3 muscle groups. It has been shown that there is an inverse relationship between fiber CSA and maximal oxygen consumption [111, 112]. Therefore, these results suggest that DT has an important oxidative modulating effect on the M. pectoralis, probably by imposing “endurance like” exercise on that muscle group, whereas the M. vastus lateralis is most probably more subjected to “power training” during DT. At first sight it seems not logical that de M. pectoralis increases its oxidative capacity through an increased amount of type I fibers and at the same time importantly increases its muscle diameter, an effect that is expected to occur in response to power training. On the other hand the M. vastus lateralis decreased its aerobic capacity, since type I fibers decreased, however decreased muscle diameter at the same time. When looking at mean CSA of muscle fibers, we can see that it decreased after DT in the M. pectoralis. The event of muscle growth associated with a decrease in mean CSA supports occurrence of muscle hyperplasia [113–117] and upregulation of oxidative metabolic machinery [118]. Indeed, small fibers are associated with a higher partial pressure of oxygen, and thus aerobic processes can easily take place in these fibers [118]. Although the M. vastus lateralis decreased in muscle diameter, the mean CSA did not change in response to DT. Therefore, the decrease in muscle diameter should not be viewed as muscle atrophy, however rather as the consequence of disappearing intramuscular fat stores. The evolution in muscle fiber type composition shows that DT induces a shift in phenotype that resembles power training in the M. vastus lateralis, which coincides with the upregulation of the glycolytic machinery identified with untargeted metabolomics in the current study.

Up till now, there are no data available on the minimum training duration required to induce shifts in muscle fiber type composition in horses. A study of Eto et al. (2004) could not detect significant changes in myosin heavy chain composition after 12 weeks of high intensity training of Thoroughbred horses [119]. But 16 weeks seemed to be enough to effectively decrease type IIX and increase type IIA fibers in Thoroughbred horses [39]. It is important to keep in mind that shifts in muscle fiber type composition are expected to be accompanied with shifts in the metabolic fingerprint of a certain muscle, although obviously, from a physiological point of view it can be assumed that metabolic shifts precede muscle fiber type shifts, which means that even if fiber type composition of a muscle does not change visually with the conventional immunohistochemical myosin heavy chain staining protocol, the shift in metabolic machinery most probably has already taken place. It can be expected that both of them do not reach their optimal configuration at the same time, however, the time lag between both phenomena is unknown.

### The effect of 8 weeks of DT on muscle metabolic profile of M. pectoralis and M. vastus lateralis

The currently widely applied conventional tools to obtain a view on the physiological adaptations of the equine muscle to training, such as muscle fiber typing, assessment of glycogen content and enzymatic activity, all have well recognized limitations [111, 120, 121]. The current study has revealed the involvement of several unnoticed metabolites that deserve future attention in horses, such as acylcarnitines, BCAAs, AAAs and specifically for the Friesian breed: glycine and proline metabolism.

Corresponding to the different muscle fiber type shifts seen in the current study for the M. pectoralis and the M. vastus lateralis in response to training, different metabolic shifts were also observed for both monitored muscles. It is important to realize that the current study pertained to resting biopsies and that no biopsies were harvested after acute exercise. These results represent thus “a local view” in a “local muscle”. In general, the machinery for fatty acid oxidation was significantly upregulated in the M. pectoralis that also showed plasticity towards a more pronounced slow twitch profile in response to 8 weeks of DT; versus a significant increased readiness of the machinery for glycolysis, pentose phosphate pathway activity and BCAA catabolism that was seen in the M. vastus lateralis, which showed plasticity towards a more pronounced fast twitch profile. Important to notice is the lack of change in short chain fatty acid metabolism and the modest change in glycogen metabolism pathway only in the M. vastus lateralis. Below, a detailed overview is provided, each time focusing on a specific metabolic pathway, looking into the effect of 8 weeks of DT, followed by comparing both M. pectoralis and M. vastus lateralis.

#### Fatty acid oxidation is significantly upregulated in response to 8 weeks of DT in the M. pectoralis

Fatty acid metabolism entails on one hand catabolic processes that generate ATP, and on the other hand anabolic processes that generate important molecules such as phospholipids that are important building blocks for all cell membranes, second messengers, local hormones and ketone bodies. In the current study the fatty acid oxidation (catabolism) pathway was significantly upregulated in response to 8 weeks of DT in the M. pectoralis. This was significantly less pronounced in the M. vastus lateralis. Lipids, which are predominantly stored in fat depots inside the body, are composed of one glycerol molecule and three free fatty acid molecules. These free fatty acids can be either labeled as short chain fatty acids (their aliphatic tail contains less than 5 carbons); medium chain (contain in between 6 and 12 carbons) or long chain (contain 13 to 21 carbons).

It is well known that fatty acids generate the largest amount of ATP, when compared to other fuels such as carbohydrates, proteins and ketones. Indeed, one mole of carbohydrates yields 36 to 38 moles of ATP, whereas, depending on the type of fatty acids involved, one mole can yield more than 450 moles of ATP [122]. Due to the complexity of the oxidative burning of fats, this pathway does not generate ATP at high speed, that is why it can only provide ATP for realization of low grade exercise of long duration, in other words, aerobic exercise. This is because oxidative burning of fats encompasses several different successive steps (Fig 1). First the fatty acids need to be “activated” by coupling them to acetyl-CoA in the cytosol of the muscle cell. Thereafter, they are shuttled by acylcarnitines through the inner and outer membrane of the mitochondria, right into the mitochondrial matrix [123, 124]. Inside the mitochondrial matrix, first β-oxidation takes place, yielding acetyl-CoA that is subsequently drawn into the TCA cycle. The TCA cycle produces FADH_2_ and NADH + H^+^, which are subsequently drawn into the electron transfer system to produce a large amount of ATP. After 8 weeks of DT, multiple long chain fatty acids (LCFAs) were significantly decreased in the M. pectoralis (i.e. saturated fatty acids palmitate, stearate, arachidate; and unsaturated fatty acids palmitoleate, 10-heptadecenoate) together with a significant increase in long chain acylcarnitines, which are the carnitine-bound forms of LCFAs necessary for the transport of LCFAs into mitochondria, demonstrating upregulation of fat oxidation pathways. These findings are in accordance with a study of Garvey et al. (2015) that applied untargeted metabolomics on soleus and plantaris muscle of rats after 8 weeks of voluntary treadmill running. They found a significant reduction in LCFAs and a significant increase in acylcarnitines [21]. In contrast, Klein et al. (2020) found both increased LCFA content and increased long chain acylcarnitines in the M. gluteus medius of horses after an aerobic training period of 12 weeks [23]. Our study results show that the full β-oxidation machinery of the M. pectoralis has been lifted to a higher level of readiness, after 8 weeks of DT. In humans, it has also been shown that decreased concentrations of LCFAs in the skeletal muscles are associated with higher levels of insulin sensitivity and an increased glycogen storage capacity [125, 126]. The significant increase in type I muscle fibers, together with the significant decrease of mean CSA seen in the M. pectoralis after 8 weeks of DT, are in line with these results. Type I fibers are well known for their profound aerobic capacity and rely thus entirely on aerobic pathways such as β-oxidation to generate energy (Fig 1).

Only long chain (LCFAs) and no medium chain fatty acids (MCFAs) were found in the present study, which is in accordance with two other studies that looked into fat composition of equine muscles, in which they could not find MCFAs and concluded that fat of equine muscle is made predominantly out of LCFAs C16 and C18 [96, 127]. Important to notice is that no significant changes were detected in the level of the short chain fatty acids (SCFAs) such as butyrate and valerate. This brings to question whether these are important fuels for horses. Also, in the study of Klein et al. no changes in SCFA levels could be detected [23]. Several equine studies have suggested in the past that SCFAs most probably account for 70 to 80% of energy needs [128–130]. However, based on the current study results, one could question this from a physiological point of view. In ruminants, SCFAs produced by ruminal flora, are the most important energy source [131], however, in horses, most probably this is not the case and other microbiome related xenobiotic metabolites may be of much greater importance to fuel the TCA cycle. It could be an option, for example, that BCAAs are produced by the intestinal flora in horses [23, 132]. Indeed, recent findings have demonstrated a positive correlation between microbiome composition and BCAA blood levels in both humans [133] and horses [132].

#### Glycolytic pathways were significantly upregulated in response to 8 weeks of DT in the M. vastus lateralis

In line with the decrease in type I muscle fibers seen in the M. vastus lateralis after 8 weeks of DT, metabolomics show a significant upregulation of the anaerobic glycolytic machinery in that muscle. Glycolysis is the first step that takes place in the carbohydrate catabolism in the cytosol of the muscle cell (Fig 1). Glycogen, that functions as a carbohydrate storage fuel inside the cytosol, is broken down up until the level of pyruvate, rendering rather low amounts of ATP. For reference, the conversion of 1 mole of glucose into pyruvate yields 2 moles of ATP [122]. When anaerobic metabolism prevails, pyruvate will not be drawn into the mitochondria to step into the TCA cycle and produce important amounts of ATP; instead, the pyruvate is converted to lactate by the enzyme lactate dehydrogenase (LDH). The anaerobic glycolysis can deliver quickly ATP necessary for explosive exercise, however, this motor can only function for a short amount of time, since it swiftly consumes all stored glycogen and it coincides with the accumulation of lactate. Metabolomics show upregulation of glycogen breakdown products and upregulation of both early (glucose, glucose-6-phosphate and fructose-6-phosphate) and late-stage (pyruvate and lactate) glycolytic intermediates (Fig 1). Especially the pyruvate increase of 3.01 fold was quite striking. Part of the pyruvate will be transaminated to alanine, which is also in line with the significant increase in alanine seen in the M. vastus lateralis. The TCA cycle was not upregulated and lactate levels were increased, all of which support the upregulated anaerobic machinery of the M. vastus lateralis in response to 8 weeks of DT. All of this was not seen in the M. pectoralis muscle.

#### TCA cycle was upregulated in M. pectoralis after 8 weeks of DT

When comparing trained (DT)/untrained metabolomics profile of the M. pectoralis to the M. vastus lateralis, almost all intermediates of the TCA cycle were increased, which is in line with the upregulation of β-oxidation of fats that produces acetyl-CoA, which on its turn is drawn into the TCA cycle. This is not the case for the M. vastus lateralis. Still, there are two metabolites of the TCA cycle that are significantly increased in both M. pectoralis and M. vastus lateralis to quite an extent, namely succinylcarnitine and 2-methylcitrate. It is possible that the TCA cycle is “fed” by other products than pyruvate that jump into the cycle at steps further downstream from acetyl CoA and not at the top, which is the obvious port of entry for pyruvate. A possible candidate for such scenario are the BCAAs and other microbiome derived xenobiotics (Fig 1) [23, 132, 134–136]. It is well known that BCAA are catabolized, especially during exercise, to acetyl-CoA and/or succinyl-CoA, which supply the TCA cycle [23, 135, 137]. BCAAs serve thus as energy sources and substrates to expand the pool of TCA cycle intermediates [135]. Microbiome derived xenobiotics could be as well a possible candidate, since evidence exists showing that gut microbiome composition can influence the structure, function and energy expenditure of muscles [134]. However, how these xenobiotics reach the muscles and how their catabolism takes place has still to be unraveled.

From a physiological point of view these alternative fuels, feeding the TCA cycle, or even directly feeding the OXPHOS system [138], are expected to be processed very quickly since they do not surpass all cycle stadia and they are expected to be glycogen sparing as has been shown for BCAAs in several studies [139–142]. Most probably these fuels fit best with oxidative fast twitch type IIA fibers, the predominant muscle fiber type of horses. Important to notice is the modest change in the glycogen metabolism pathway and even only in one muscle group (M. vastus lateralis) in response to 8 weeks of DT. Even if the training protocol used in this study was not sufficient to induce glycogen depletion, these results bring to question as to whether glycogen needs to be viewed as the most important energy source in horses. In contrast to human who need 24h to replenish their muscle glycogen content, horses need 48 to 72h. Physiologically this does not comply with what would be expected from an essential energy source [24].

#### The pentose phosphate pathway (PPP) was significantly upregulated in response to 8 weeks of DT in the M. vastus lateralis

The PPP takes place in the cytosol and has several different functions (Fig 1). From a phylogenetic point of view it is very old and probably dates back to the prebiotic world. It contains two distinct phases: a first oxidative phase, which is irreversible and results in the production of NADPH. One of the main goals of NADPH in the cell is to reduce oxidative stress via reduction of glutathione. Besides that, NADPH oxidizes pyruvate to malate, which is an intermediate of the TCA cycle. NADPH is also involved in fatty acid synthesis. The second step of the PPP is the non-oxidative production of ribose-5-phosphate necessary for production of nucleotides, nucleic acids and recycling back to fructose-6-phosphate; the latter can be drawn into the anaerobic glycolysis cycle and produces erythrose-4-phosphate necessary for the production of AAAs. Especially ribulose/xylulose-5-phosphate and fructose-6-phosphate were significantly upregulated in the M. vastus lateralis to a striking extent (respectively 4.00 fold and 1.70 fold). This was not the case for the M. pectoralis. Whether or not the PPP plays an important role as additional cycle to fuel the anaerobic glycolysis by furnishing it with fructose-6-phosphate, is not known in horses. In humans and rats, the PPP has a predominant anabolic function and is especially active in tissues with rapidly dividing cells such as bone marrow, skin and gastric mucosa, in need of high rate production of nucleotides [143–145]. With that respect, ribulose/xylulose-5-phosphate are viewed as the rate limiting intermediates for the de novo synthesis of nucleotides [146]. Fast-twitch muscle fibers are known to realize a much higher rate of nucleotide synthesis when compared to slow-twitch fibers, due to the high speed at which fast-twitch processes take place [146]. Therefore, the upregulation of the PPP in the M. vastus lateralis after 8 weeks of DT matches with the muscle fiber type composition switches seen in that muscle.

#### Amino acid metabolism was significantly influenced in response to 8 weeks of DT

Skeletal muscle is considered to be the largest protein pool inside the body. Any type of training, whether it is resistance training or endurance training, is expected to have its impact on skeletal muscle amino acid metabolism, pushing the balance towards anabolic activity, in support of muscle build-up [147–149]. With that respect, BCAAs were upregulated in the M. vastus lateralis, whereas glutamine/glutamate metabolism was upregulated in the M. pectoralis. For both muscle groups, there was a significant upregulation of AAAs, glycine metabolism and xenobiotic metabolism.

*Branched-chain amino acids (BCAAs) were upregulated in response to 8 weeks of DT in the M*. *vastus lateralis*. The class of BCAAs is represented by three essential amino acids: valine, leucine and isoleucine (Fig 2). They represent about 35% of the essential amino acids in the muscle [150, 151]. What distinguishes them from other amino acids is the fact that they are non-polar and their R-group is a branched chain. The fact that they are essential means that they need to be ingested by the diet, at least in human and many other species [152]. In horses, it is not known, though their vegan diet for sure serves as a source. A study in cows has demonstrated that gut microbiome production of BCAAs is important as well [153].

In contrast to many other amino acids, breakdown of BCAAs does not take place predominantly in the liver, due to low hepatic activity of branched-chain amino acid aminotransferase (BCAT). As a consequence, BCAAs supplemented orally are available for many extrahepatic tissues, such as muscle tissue. Positive effects of BCAA supplementation have been reported in a wide array of human and animal studies focusing on mitigation of cachexia and muscle wasting, suppression of symptoms of encephalopathy, promotion of wound healing and with respect to exercise physiology: attenuation of muscle fatigue and stimulation of insulin release [154–157]. BCAAs can function as fuel to generate ATP to perform exercise. They enter the muscle cell via transmembranar transportation molecules, such as L-type amino acid transporter 1 (LAT1) and LAT2 [158–160]. BCAA catabolism is an oxidative process of which the breakdown products are fed into the TCA cycle at steps further downstream from acetyl CoA (Figs 1 and 2). The total oxidation of one mol of respectively leucine, isoleucine and valine generates 43, 42 and 32 moles of ATP. From an energetic point of view, it is thus an interesting pathway. It has been shown that leucine oxidation is greater in trained rats when compared to untrained rats [161, 162]. BCAA breakdown occurs in three consecutive steps. The first step is catalyzed by the enzyme BCAT, which deaminates the three BCAAs into their respective α-keto-branched-chain acid, being α-ketoisovalerate for valine; α-keto-β-methylvalerate for isoleucine and α-ketoisocaproate for leucine. Subsequently, a large multi-enzyme complex situated on the inner mitochondrial membrane, known as branched-chain α-ketoacid dehydrogenase (BCKDH) converts these α-keto-branched-chain acids in two consecutive steps into products such as succinyl-CoA and acetyl-CoA, which are intermediates of the TCA cycle, succinyl-CoA being fed into the TCA cycle at steps further downstream from acetyl CoA. Both biotin and vitamin B_12_ are important co-factors for those steps. The shuttling of BCAAs into the mitochondria is performed by acylcarnitines [163]. Both BCAA and glycyl hydropeptides of BCAAs were significantly increased in the M. vastus lateralis, supporting the idea that 8 weeks of DT upregulates readiness of the BCAA machinery in a muscle that develops a fast twitch profile. With that respect, also the significant increase of succinylcarnitine (1.4 fold change) and 2-methylcitrate (1.77 fold change in the M. pectoralis and 2.26 in the M. vastus lateralis), two entry ports for BCAA breakdown products into the TCA cycle is striking. Nothing is known about the role of BCAAs in the energy metabolism of horses and in the face of energy partition, it is not known at which time point of exercise this machinery engages. It has been shown that BCAA oxidation is higher in muscles containing a high amount of aerobic fibers compared to muscles made predominantly out of anaerobic fibers [164]. Keeping in mind the fact that horses predominantly are constituted out of aerobic fast twitch fibers, BCAAs could be a very important energy source that has gone unnoticed up until now. More research is needed with that respect. Looking back into equine literature, BCAAs have been found in several studies in muscle tissue, but their role in energy supply has been minimized probably due to the lack of knowledge of BCAA metabolism and their contribution to energy needs [62, 165, 166]. Our results are in accordance with Klein et al. 2020, who also showed that BCAA breakdown occurred significantly in equine muscle during acute exercise and that after 12 weeks of a conditioning training program (4 days/week aerobic treadmill exercise and 1 day/week high speed treadmill exercise), the BCAA content increased in resting muscle biopsies of the M. gluteus medius [23].

*Glutamine/glutamate metabolism was significantly upregulated in the M*. *pectoralis*. Glutamine content was significantly increased (1.46 fold) in the M. pectoralis. Several studies have demonstrated that there is an important positive correlation between muscular glutamine levels and the muscular protein synthesis balance [167, 168]. An upregulated anabolic profile is in accordance with the increased muscle diameter of the M. pectoralis seen after 8 weeks of DT. Glutamine shuttles nitrogen between tissues and is involved in several metabolic processes such as cellular proliferation, acid–base balance and antioxidant synthesis (i.e. synthesis of GSH) [169]. Exhaustive exercise and starvation caused glutamine deficiency [170–172]. Similarly, muscle glutamine levels declined in overtrained individuals [173]. Glutamine is known to be an important C donor for gluconeogenesis and glycogen synthesis [174–176]. More recent studies have demonstrated a positive modulating effect of glutamine on insulin sensitivity and glycemic control [177–181]. Physiologically, these are all beneficial effects for a muscle that develops a more pronounced aerobic profile, such as the M. pectoralis in response to 8 weeks of DT [177–181]. Finally, also N-acetyl-aspartyl-glutamate (NAAG) was upregulated in the M. pectoralis DT group. NAAG is the most important neurotransmitter in the mammalian brain and might have neuroprotective properties [182].

*Aromatic amino acids (AAAs) were significantly upregulated in both muscles*, *predominantly the M*. *vastus lateralis following 8 weeks of DT*. AAAs, which have an aromatic ring such as tryptophan, tyrosine, phenylalanine and histidine were all significantly upregulated predominantly for the M. vastus lateralis. Among this group, three are essential: phenylalanine, tryptophan and histidine. Especially the upregulation of derivatives of histidine is striking: cis-urocanate (4.75 fold), carnosine (1.10 fold) and homocarnosine (1.40 fold) in the M. vastus lateralis. Cis-urocanate is an intermediate of the histidine degradation pathway. It is synthetized from histidine by uncoupling ammonia. Further degradation leads to production of glutamate, which on its turn is important for BCAA breakdown. Carnosine, is a dipeptide consisting of the amino acids β-alanine and histidine. It is found in large amounts in muscle and brain tissue. It is known to mitigate acidosis due to its buffering capacity, to act as an anti-oxidant, and to improve excitation-contraction coupling by regulating Ca^2+^ fluxes in the sarcoplasmatic reticulum [183]. Several studies have shown that carnosine supplementation improves performance capacity, especially for high intensity exercise [184–188]. Interestingly, human training studies show very little effect of training on muscle carnosine content. High carnosine levels are reported to be genetically determined or to occur as long term adaptation in response to years of training [189]. Apparently, this does not apply for horses, which can probably be attributed to their pronounced fast twitch profile.

Tyrosine (1.23 fold) and tryptophan (1.24 fold) were both upregulated in the M. vastus lateralis. In several studies the muscular content of tyrosine is used as an indicator of muscular protein catabolism, since the muscle cannot metabolize this amino acid [190, 191]. A rat study has shown that tyrosine levels remain high in the muscles for more than 24h after a swimming experiment of 10h duration [192]. Tryptophan is an important building block for serotonin production. In human athletes, the plasma tryptophan/BCAA ratio is monitored for early detection of occurrence of central fatigue [193] and tryptophan supplementation is known to postpone the occurrence of central fatigue when performing endurance exercise [194]. Tryptophan also functions as a precursor for the kynurenine pathway, which is a complex metabolic pathway that generates NAD^+^, which obviously is involved in many metabolic processes. Increased plasma levels of tryptophan have been reported after prolonged exercise such as military training or marathon races [195]; triathlons [196] and more than 4h of cycling [197]. Tryptophan can also exert a mitigating effect on the muscular inflammatory response. A recent study showed that low intensity aerobic exercise associated with oral supplementation of tryptophan in rats with fibromyalgia diminished the pro inflammatory cytokine IL-6 release in muscles, as well as serum cortisol levels [198]. It is not known whether tryptophan can mitigate the training induced inflammatory response in healthy subjects.

P-cresol-glucuronide was also importantly upregulated in both muscles (2.69 fold in M. pectoralis and 2.91 fold in M. vastus lateralis). This metabolite is the result of bacterial fermentation of dietary tyrosine [199]. So, most probably, it needs to be viewed as another xenobiotic, just like the previously reported BCAA dipeptides, that is produced by the microbiome and is used inside the muscle as fuel source [23].

*Glycine and serine metabolism was significantly upregulated in response to DT in both M*. *pectoralis and M*. *vastus lateralis*. Glycine is a very important amino acid and can be synthesized out of glucose, glutamate, betaine, serine, threonine, choline, and hydroxyproline. Several of these building blocks were significantly upregulated in both muscles after 8 weeks of DT. Also serine showed an important upregulation in both muscles (1.45 fold in M. pectoralis and 1.89 fold in M. vastus lateralis). Notably, serine can be converted to pyruvate which can then be further processed by anaerobic glycolysis, or drawn into the TCA cycle in case aerobic metabolism prevails. Sarcosine, which was also significantly upregulated in both muscles (2.22 fold in M. pectoralis and 3.10 fold in M. vastus lateralis) is an intermediate of the choline-supported pathway of glycine synthesis. Glycine is crucial for a series of important metabolic processes such as synthesis of proteins, glutathione, heme, creatine, nucleic acids, and uric acid and gluconeogenesis. Interesting to note is that glycine accounts for 1/3 of amino acids in collagen and elastin. Keeping in mind, previous publications, in which a standard upregulation of collagen breakdown in healthy Friesian horses was reported, it would be interesting to check whether a similar upregulation of glycine metabolism is seen in other horse breeds in response to 8 weeks of DT [200]. In the study of Klein et al. (2020) no changes in glycine or proline metabolism were reported for the Standardbreds supporting the concept that this a Friesian specific trait [23]. These findings are also in accordance with previous studies of our research group focusing on aortic rupture and mega esophagus in Friesian horses, two important hereditary diseases in this breed, most probably expressed on top of an aberrant collagen and elastin metabolism [200–203].

*Methionine and cysteine metabolism were upregulated in response to 8 weeks of DT in both muscles*. Especially intermediates of cysteine and methionine metabolism were significantly increased in both muscles. Methionine, which is an essential amino acid, functions as a precursor for cysteine, succinyl-CoA, homocysteine, cysteine, creatine, and carnitine. Methionine has important mitigating effects on metabolism, such as stimulation of protein synthesis and increasing the capacity to cope with oxidative stress. The increased glutathione levels, found in both muscles support this. It is well known that training improves the antioxidant defense mechanisms in order to cope with higher oxidative stress levels [204, 205].

*Proline and arginine metabolism were significantly upregulated*, *especially in M*. *pectoralis*, *after 8 weeks of DT*. Proline and arginine are biosynthesized out of glutamate, which was also upregulated in the M. pectoralis (1.19 fold). Also ornithine, which is an important metabolite of the urea cycle, was significantly upregulated (2.02 fold in M. pectoralis and 1.33 fold in M. vastus lateralis). These findings support an upregulation of protein metabolism in especially the M. pectoralis. Interestingly, in our study arginine intermediates were also upregulated, which can function as precursors for ornithine synthesis. Ornithine is important in the urea cycle, since it binds the ammonia group and is than recycled to start the urea cycle again.

Proline was significantly upregulated in both muscle groups (1.16 fold in M. pectoralis and 1.39 fold in M. vastus lateralis) and is known to be a biomarker for collagen content in muscles of dogs [206]. In view of the previous remarks concerning collagen metabolism in healthy Friesian horses, more research is needed with that respect.

#### Glutathione metabolism was altered in response to 8 weeks of DT in both muscles

Several glutathione metabolism intermediates were significantly upregulated in both muscle groups, still more pronounced in the M. pectoralis, indicating an increased capacity to cope with oxidative stress. It is well known that training induces a low grade inflammatory reaction, but in the same time induces protective mechanisms against oxidative stress, especially in muscles that undergo aerobic training [207–209].

Possible limitations of the current study were the fact that no comparable follow-up was performed in a third group of horses, housed in a similar fashion, but without being trained, to discern between pure training effects and effects that could have manifested themselves naturally in horses of 2.5 to 3.5 years old over the course of 8 weeks. Secondly, for allowing a comparative approach, DT was just like AT performed at a speed of 1.25 m/sec which is a rather low training intensity.

## Conclusion

AT is superior to DT to increase muscle diameter in the hindquarters, with maximum effect reached already after 4 weeks for some muscle groups. DT decreased muscles of the hindquarters and increased muscles of the forehand, again with maximum effect reached after 4 weeks for some muscle groups.

Type IIA fibers were the predominant muscle fiber type in all studied muscles. The M. semitendinosus contained less type I fibers when compared to the M. pectoralis and the M. vastus lateralis, both of which showed similar muscle fiber type composition. The mean fiber CSA of the M. pectoralis and the M. semitendinosus was significantly larger than that of the M. vastus lateralis and CSA of type I fibers was significantly larger in the M. pectoralis when compared to the M. vastus lateralis.

Different physiological adaptations occurred in the monitored muscle groups in response to the same type of training (Table 3). The M. semitendinosus showed only minor changes, which proves that it is important which muscle groups are selected for longitudinal follow-up in training trials. After DT, the M. pectoralis showed increased muscle diameter, more type I fibers, decreased mean fiber CSA, and an upregulated oxidative metabolic profile: increased β-oxidation (key metabolites: decreased long chain fatty acids and increased long chain acylcarnitines), TCA activity (intermediates including succinyl-carnitine and 2-methylcitrate), amino acid metabolism (AA) (glutamine, aromatic AAs (AAAs), serine, urea cycle metabolites such as proline, arginine and ornithine) and xenobiotic metabolism (especially p-cresol glucuronide). The M. vastus lateralis expanded its fast twitch profile, with decreased muscle diameter, decreased type I fibers and an upregulation of glycolytic and pentose phosphate pathway (PPP) activity, and increased branched-chain and AAA metabolism (cis-urocanate, carnosine and homocarnosine, tyrosine, tryptophan and p-cresol-glucuronide, serine, methionine, cysteine, proline and ornithine).

**Table 3:** Overview of muscle morphometrics and muscle metabolism in horses.

Parameters			M. pectoralis	M. vastus lateralis	M. semitendinosus
**Effect of dry treadmill training on muscle morphometrics**					
**Muscle morphometrics**	Muscle diameter	Trained	**increased**	**decreased**	**decreased**
	Fiber type composition	Untrained	type I M. pectoralis = M. vastus lateralis < M. semitendinosus		
		Trained	**type I**	**type I**	unchanged
	Mean CSA	Untrained	M. pectoralis = M. semitendinosus > M. vastus lateralis		
		Trained	**decreased**	unchanged	unchanged
	Fiber CSA	Untrained	type I M. pectoralis > M. vastus lateralis		not significant
		Trained	unchanged	unchanged	unchanged
**Effect of dry treadmill training on metabolic profile (fold change)**					
**Energy pathways**	FA oxidation	Long chain FAs	**0.2–0.7**	unchanged	
		Long chain acylcarnitines	**1.35–2.21**	**1.41–1.85**	
	TCA cycle	Succinylcarnitine	**1.40**	**1.43**	
		2-Methylcitrate	**1.77**	**2.26**	
	Glycolysis	Pyruvate	unchanged	**3.01**	
	PPP	Ribulose/xylulose -5-phosphate	unchanged	**4.00**	
	BCAA	BCAA-dipeptides	unchanged	**1.20–2.20**	
	Amino acid metabolism	Carnosine	**1.11**	**1.10**	
		Homocarnosine	**1.44**	**1.40**	
		Tryptophan	**1.19**	**1.24**	
		Tyrosine	unchanged	**1.23**	
		Sarcosine	**2.22**	**3.10**	
		Glutamine	**1.45**	unchanged	
		Serine	**1.45**	**1.89**	
		Proline	**1.16**	**1.39**	
		Cis-urocanate	**2.63**	**4.75**	
		Ornithine	**2.02**	**1.33**	
	Xenobiotics	P-cresolglucuronide	**2.69**	**2.91**	
	Gluthatione	GSH	**1.60**	**1.57**	

Effect of 8 weeks of dry treadmill training (DT) on muscle morphometrics and muscle metabolism in the M. pectoralis, M. vastus lateralis and M. semitendinosus of Friesian horses. FA: fatty acid; TCA: tricarboxylic acid; PPP: pentose phosphate pathway; BCAA: branched-chain amino acid; AAA: aromatic amino acid. In red: significantly increased; in green: significantly decreased.

The fact that only modest glycogen metabolism pathway changes were seen and no changes in short chain fatty acids brings to question whether these are pivotal energy sources for horses. Results show that BCAAs, AAAs and microbiome-derived xenobiotics need further study in horses. They feed into the TCA cycle at steps further downstream from acetyl CoA and most likely are oxidized in type IIA fibers, the predominant fiber type of the horse. These study results underline the importance of reviewing existing paradigms on equine bioenergetics.

## Supporting information

S1 Table Muscle fiber type composition and muscle fiber cross sectional area of the M. pectoralis, M. vastus lateralis and M. semitendinosus. Raw data of muscle fiber type composition and muscle fiber cross sectional area in the M. pectoralis, M. vastus lateralis and M. semitendinosus in untrained condition (untrained) and after 8 weeks of dry treadmill training (trained).
(XLSX)

S1 File Detailed overview of the analytical method of the metabolomics analysis. (PDF)
