# Supplementary material for: Flexibility of equine bioenergetics and muscle plasticity in response to different types of training: An integrative approach, questioning existing paradigms
Source: PLoS One. 2021 Apr 13;16(4):e0249922. doi: 10.1371/journal.pone.0249922 (PMC8043414; doi:10.1371/journal.pone.0249922)
Supplement: S1 File — (PDF) [file pone.0249922.s002.pdf]

Supplementary file

Sample Preparation and Metabolic Profiling

Metabolic profiling of all muscle samples was performed at Metabolon, Inc. (Durham, NC). Samples were prepared using the automated MicroLab STAR® system from Hamilton Company and proteins were precipitated with methanol. Several recovery standards were added prior to the first step in the extraction process for quality control purposes. The resulting extract was divided into five fractions: two for analysis by two separate reverse phase (RP)/UPLC-MS/MS methods with positive ion mode electrospray ionization (ESI), one for analysis by RP/UPLC-MS/MS with negative ion mode ESI, one for analysis by HILIC/UPLC-MS/MS with negative ion mode ESI, and one sample was reserved for backup.

The sample extract was dried then reconstituted in solvents compatible to each of the four methods. Each reconstitution solvent contained a series of standards at fixed concentrations to ensure injection and chromatographic consistency. One aliquot was analyzed using acidic positive ion conditions, chromatographically optimized for more hydrophilic compounds. In this method, the extract was gradient eluted from a C18 column (Waters UPLC BEH C18-2.1x100 mm, 1.7 µm) using water and methanol, containing 0.05% perfluoropentanoic acid (PFPA) and 0.1% formic acid (FA). Another aliquot was also analyzed using acidic positive ion conditions, however it was chromatographically optimized for more hydrophobic compounds. In this method, the extract was gradient eluted from the same afore mentioned C18 column using methanol, acetonitrile, water, 0.05% PFPA and 0.01% FA and was operated at an overall higher organic content. Another aliquot was analyzed using basic negative ion optimized conditions using a separate dedicated C18 column. The basic extracts were gradient eluted from the column using methanol and water, however with 6.5mM Ammonium Bicarbonate at pH 8. The fourth aliquot was analyzed via negative ionization following elution from a HILIC column (Waters UPLC BEH Amide 2.1x150 mm, 1.7 µm) using a gradient

consisting of water and acetonitrile with 10mM Ammonium Formate, pH 10.8. In addition, several controls were analyzed in concert with the experimental samples: aliquots of a well-characterized human plasma pool served as technical replicates throughout the data set, extracted water samples served as process blanks, and a cocktail of standards spiked into every analyzed sample allowed monitoring of instrument performance. The 36 muscle samples were run in the same batch and injections were randomized.

For UHPLC/MS/MS^2 analysis, aliquots were separated using a Waters Acquity UPLC (Waters, Millford, MA) and analyzed using a Thermo Scientific Q-Exactive high resolution/accurate mass spectrometer interfaced with a heated electrospray ionization (HESI-II) source and Orbitrap mass analyzer operated at 35,000 mass resolution.

The MS instrument scanned 70-1000 *m/z* and alternated between MS and MS^2 scans using dynamic exclusion. Metabolites were identified by automated comparison of the ion features in the experimental samples to a reference library of chemical standard entries that included retention time, molecular weight (*m/z*), preferred adducts, in-source fragments, and associated MS spectra, and were curated by visual inspection for quality control using software developed at Metabolon. More than 3300 commercially available purified standard compounds are registered into Metabolons' library. Additional mass spectral entries have been created for structurally unnamed biochemicals, which have been identified by virtue of their recurrent nature (both chromatographic and mass spectral).

Instrument variability was determined by calculating the median relative standard deviation (RSD) for the internal standards that were added to each sample prior to injection into the mass spectrometers. Overall process variability was determined by calculating the median RSD for all endogenous metabolites (i.e., non-instrument standards) present in 100% of the matrix samples, which are technical replicates of pooled samples. Values for instrument and process variability were 3% and 6%, respectively.

For the data normalization, each compound was corrected in run-day blocks by registering the medians to equal one (1.00) and normalizing each data point proportionately (termed the “block correction”).
